# Comprehensive AI framework for automated classification, detection, segmentation, and severity estimation of date palm diseases using vision-language models and generative AI

**DOI:** 10.3389/fpls.2025.1710188

**Published:** 2025-12-17

**Authors:** Abid Iqbal

**Affiliations:** Department of Computer Engineering, College of Computer Sciences and Information Technology, King Faisal University, Al Ahsa, Saudi Arabia

**Keywords:** vision-language models (VLMs), generative adversarial networks (GANs), PaliGemma2, zero-shot segmentation, severity prediction, sustainable crop management

## Abstract

The date palm (*Phoenix dactylifera* L.) is a vital crop in arid and semi-arid regions, contributing over $13 billion annually to the global economy. However, it faces significant yield losses due to pests, such as the red palm weevil, and diseases, including Bayoud and Black Scorch. Currently, expert visual inspection is the primary method of management, but it is time-consuming, subjective, and unsuitable for detecting large-scale or early-stage damage. Automated approaches based on classical machine learning offer limited improvements due to their lack of generalizability and environmental sensitivity. Recent deep learning methods, such as CNNs and Vision Transformers, have improved classification accuracy, but treat tasks like classification, detection, segmentation, and severity estimation as separate. This paper proposes an integrated Reveal-Aware Hybrid Vision-Language and Transformer-based AI framework that combines GAN-based augmentations for feature generation, CLIP for multimodal classification, PaliGemma2 for text-based detection, Grounding DINO + SAM 2.1 for zero-shot segmentation, and a Vision Transformer regression model for severity prediction. This end-to-end explainable diagnostic pipeline achieved 98% classification accuracy, 95.8% precision, 91.3% recall, and 94.2% F1-score across two datasets: nine classes of infected date palm leaves and three classes of date palm diseases. The proposed framework demonstrated detection accuracy of 94-98%, high-quality segmentations, and reliable severity estimates. This integrated approach highlights the potential of combining AI, vision-language models, and transformers for scalable, accurate, and sustainable plant disease management.

## 1 Introduction

Date palm (*Phoenix dactylifera* L.) is one of the most important economic and cultural contributions to arid and semi-arid land resource systems, particularly in the Middle East, North Africa, and South Asia. This highly drought-tolerant species is a vital source of nutrition, income, and ecosystem stability for several million people in these regions. The global date fruit industry is valued at over $13 billion annually, benefiting entire communities through all aspects of cultivation, processing, and export-related markets. Unfortunately, this ring of vital crops lost productivity and potentially lost their survival. These crops are threatened by a range of biotic stressors that increasingly affect the date palm, especially in cultivated production. The red palm weevil (Rhynchophorus ferrugineus) has been one of the most damaging forms of biotic stress for the date palm. This invasive beetle has disseminated across infested territories, causing devastation to date palm forests, with some completely losing their standing palms in the process.

Every bit as detrimental as the Red Palm Weevil are several joint and solitary pathogens, especially among the fungi, such as Fusarium oxysporum f. sp. albedinis (the pathogen responsible for Bayoud disease). Thielaviopsis punctulata (which causes Black Scorch disease (Ahmed and Ahmed, 2023). These pathogens spread rapidly through the forest system, killing trees through advancing, systemic infections that involve wilting and necrotic tissue, ultimately leading to the tree’s death. Fungal infections are further complicated because they can spread so rapidly to adjacent trees through soil and water. Pathologically, oomycetes such as the Bayoud disease pathogen have killed more than 12 million date palms in North Africa since the first detection of the species. The economic impact is overwhelming, as estimated losses to affected areas exceed $800 million annually, associated with decreases in yield (40-70% decreases in heavily infected areas) and increased expenditure on pesticides and other pesticides as the government implements tree removal programs. Standard disease management relies on agriculture extension workers visually identifying disease symptoms, which is limited by human subjectivity and the time required to train experts (as opposed to agriculture extension workers). None of the available disease management approaches can determine when the disease is in the early stages of infection, which would allow for the most effective intervention (Kamal et al., 2025). Even if agriculture extension workers do pick up on severe sufferers from infections, they only inspect one date palm tree, rather than a small number or larger experiment along the overall palm tree plantation, which is labor intensive and as a result would still not create conditioned monitoring across large plantation areas, thus again allowing infection to spread openly that would only be identified when the palm tree has undergone permanent irreversible destructive changes (Nadeem et al., 2025). The current state of limited coverage in detecting the majority of disease symptoms requires innovative approaches driven by technology that will enable accurate and reliable diagnoses to be made rapidly and at scale. The ideal system is an automatic diagnostics platform that can incorporate multiple diagnostics dimensions, including differentiating types of diseases, locating infected tissues, quantifying the severity of infection, and providing treatment recommendations, all in the field under real-world conditions. Regardless of whether a single automated diagnostic triggers a trajectory for developing a multifaceted diagnostics platform as outlined above, this development will transform date palm brown rot and unique best management practice protocols in terms of crop protection and/or avoidance of agricultural losses in the billions and thereby resolve excess pesticide use with treatment coupled to identification of infection amounting to protestations of these losses, along with crop sustainability for future generations (Namoun et al., 2024). Historically, date palm disease management began with traditional methods that depended on the knowledge of trained agronomists and farmers. The evaluation of the date palm was conducted by examining the leaves, trunks, or fruits to identify symptoms of illness, such as blemishes, wilting, or the presence of insects (Arafat et al., 2025). Although they rely on a practical knowledge base, traditional disease management methods for date palm disease are slow, labor-intensive, and prone to human error. Furthermore, conventional methods are only beneficial when visual symptoms are present, which often means that a large percentage of the crop is already damaged. Disease control measures have limited impact (Gibril et al., 2021).

The shift from manual inspection to automation signified progress in the ability to detect date palm diseases using images, with early developments using traditional machine learning (ML). These early efforts employed traditional computer vision algorithms to extract handcrafted features, which included texture features (e.g., Local Binary Patterns, Grey-Level Co-occurrence Matrices), histograms of color, and geometric features (Hessane et al., 2025). These features were evaluated using classical classifiers (e.g., Support Vector Machines (SVM), K-Nearest Neighbors (KNN), and Random Forests (RF)) to distinguish between healthy and sick palms (Hessane et al., 2023b). This approach represented an improvement over a subjectively driven human evaluation, but the overall approach had some significant limitations. First, the performance of these models depended on the quality and discriminating power of the engineering features. These engineered features, for the most part, did not accurately represent the set of potential visual patterns for early-stage infections with date palm diseases. Second, when tested in new environments, the quality of the derived features significantly degraded, depending on the lighting or camera angle, which further limited their use in a classification context. Third, it had a poor ability to generalize due to intra-class variability in disease symptoms and inter-class resemblance of diseases, which ultimately resulted in an excessive number of false positives and unreported damages. Moreover, the feature extraction process itself was computationally intensive, and domain knowledge was required to create high-quality feature sets for various diseases, rendering the system inflexible and making it difficult to adapt to novel pathogens or changing environmental conditions. The drawbacks of this approach highlighted the need for more robust and adaptive approaches that could automatically learn to extract discriminative features from data, leading to the emergence of proposed deep learning techniques for agricultural disease detection. A major transition occurred when deep learning (DL) and, more notably, convolutional neural networks (CNNs) gained widespread use. CNNs introduced a revolutionary automated feature extraction method that learns complex hierarchical representations from raw pixels (Safran et al., 2024). This shift enabled models to be much more robust and accurate. The research community has since adopted and deployed various CNN architectures, including pre-trained models such as VGG, ResNet, and InceptionV3, to classify date palm diseases, resulting in high accuracies (Sagheer et al., 2025). Recognized architectures, such as YOLO, have also been utilized for object detection in infected areas by visually representing them within bounding boxes (Nadeem et al., 2025). Nevertheless, both methods are highly effective for tasks but do not lend themselves to applications such as classification, detection, and segmentation that require the use of separate models, which can be computationally and operationally expensive. Building on CNN’s success, transformer-based models represent another potential path forward. Initially applied to natural language processing (NLP), Vision Transformers (ViT) and their close relatives, such as Swin Transformers, utilize a self-attention architecture on image data, allowing them to learn long-distance dependencies among pixel values and achieve both global and local understanding of the context of an entire image (Sagheer et al., 2025). These advantages can be significant for recognizing the presence of complex, distributed, and/or atypical disease patterns that CNNs may overlook. However, these models are still fundamentally unimodal vision-only models that recognize only what is in the visual data and can’t incorporate or reason with text-based information, which is a rich source of information about plants and is integrally related to agronomists’ diagnostic processes (Bouthaina et al., 2024). A notable gap in the existing literature is the absence of an integrated, multi-task framework that utilizes both visual and linguistic information to deliver a more comprehensive and explainable diagnosis. The emphasis in the current literature on classification, detection, segmentation, and severity prediction as distinct tasks, which are most commonly isolated and solved with separate models, creates a lack of understanding and an inconvenient diagnostic workflow that does not include the extensive context provided by expert descriptions and annotations. Therefore, it seems logical to develop a framework that can complete all of these tasks in a single system and subsequently produce human-interpretable results. This research fills this gap by proposing a new multifaceted AI framework that incorporates the cutting-edge capabilities of advanced Vision-Language Models (VLMs) and generative AI. Specifically, a comprehensive pipeline was created, including multiple state-of-the-art models, and a chain of tasks was accurately and efficiently executed. Rather than being a simple classification, our framework is an end-to-end diagnostic and severity assessment solution. The principal innovations proposed are:

- Generative AI for Data Augmentation: I employ a diffusion-based generative model to augment rare and underrepresented disease categories in the Infected Date Palm Leaves Dataset and the Palm Disease Dataset, aiming to achieve a more balanced and diverse representation.
- CLIP for Multimodal Classification: CLIP is a vision-language model that aligns images with natural language. I use CLIP to allow for prompt-based classification. CLIP enables strong classification performance, even in a few-shot or zero-shot manner, using textual descriptions of diseases.
- PaliGemma-2 for Language-Driven Detection: I leverage PaliGemma-2, a vision-language detection model that can ground objects based on text prompts, thereby facilitating comprehensible and flexible disease localization.
- Grounding DINO + SAM 2.1 for Prompt-Based Segmentation: I incorporate Grounding DINO to parse language queries and create region proposals, and Segment Anything Model (SAM 2.1) for instance segmentations without pixel-level annotations.
- ViT Regression and Segmentation Verification for Severity Estimation: I propose a vision transformer-based regression model for severity scoring, modified with a segmentation verification module to detect inconsistencies between predicted severity and visually segmented disease areas.

## 2 Literature review

The use of artificial intelligence to detect disease in date palm trees has come a long way from the early days of machine learning and deep learning. The first research done in this area relied on image processing techniques and involved conventional computer vision techniques, where disease detection relied on handcrafted features. For example (Al-Hiary et al., 2011), and (Mostajer Kheirkhah and Asghari, 2019) showed how the analysis of texture using Local Binary Patterns (LBP) and Gray-Level Co-occurrence Matrices (GLCM), and using color histograms combined with Support Vector Machines (SVM), Random Forest classifiers, and so on, was able to discover characteristics from the image that informed date palm disease detection. They found moderate levels of success, with accuracies reported within a 75-85% variation, but there were severe limitations when it came to applicability to different environments and disease presentations.

The authors (Reddy et al., 2024) explain that the drawbacks of conventional manual visual inspections include being labor-intensive, subjective, and slow to implement for larger plantations. Due to the human aspect, manual inspections may not be reproducible, and it could be challenging to identify subtle symptoms early enough for timely intervention. Recent advances in deep learning have revolutionized plant disease detection, with Convolutional Neural Networks (CNNs) yielding significantly better results than traditional methods. For example, a CNN system for detecting Red Palm Weevil infestations was developed (Nabil et al., 2025). This system reported achieving 93% accuracy by automatically learning and identifying discriminative features from images. On a similar note, a modified VGG16 architecture for palm disease classification was reported with an accuracy of 96% on a controlled dataset (Reddy et al., 2024). These methods primarily rely on deep learning models (i.e., CNNs) to train the models, thereby eliminating/reducing the time and skill required to manually derive features used in traditional methods, while achieving high detection efficiency.

New developments have specifically incorporated object detection frameworks for disease detection. For example, Ramalingam et al. (used YOLOv3 to detect five palm diseases with a mean accuracy (mAP) of 0.89. However, object detection systems are limited by the need for numerous labelled datasets and do not utilize textual or contextual information. Vision transformers (Dosovitskiy et al., 2020) exhibit more efficient learning properties than previous systems, thanks to the use of self-attentional methods. It has also not been investigated for detecting palm diseases.

In addition to classical CNNs, the state-of-the-art deep learning models, especially the transformers, are emerging as a strong alternative. While applications specific to the Swin transformer for date palm leaf disease classification are appearing, many reports about other plant diseases illustrate that the Swin transformer is quite robust. One application of an efficient Swin transformer for general plant disease classification reported a precision of 80.14% and a recall of 76.27% on the PlantDoc datasets, which represents a significant improvement over the plain Swin-T (Liu and Zhang, 2025). Additionally, a dual-track feature fusion model incorporating the Swin Transformer for grape leaf diseases produced an accuracy of 97.6%. The precision, recall, and F1-score all achieved accuracies between 96.60% (Makhija, 2024). This suggests that the Swin transformer is capable of capturing complex features to provide high precision in plant disease classification.

In recent times, the computer vision community has adopted transformer-based architectures to build models in a manner that benefits the community as a whole from the advances seen in natural language processing. Vision transformers (ViT) for image processing and their related variants (i.e., Swin transformers) utilize self-attention mechanisms, which enable them to capture global dependencies in an image, thereby providing a more comprehensive view of disease patterns from images than the patch-style approach with a receptive field in CNNs (Sagheer et al., 2025). Reported studies have shown that vision transformers achieve state-of-the-art results on plant disease classification and segmentation tasks, suggesting further promise for addressing some of the limitations of CNNs (Ahmed and Ahmed, 2023). Nevertheless, they still have significant shortcomings and fundamental limitations, as they are still susceptible to bias. They can only accept image data and do not accompany it with rich, descriptive text that retains real-world descriptors, limiting their ability to provide more comprehensive, expert-like assessments. The summary of the literature review for Palm date classification, detection, and segmentation systems is given in **Table 1**.

**Table 1:** Literature review summary for palm date classification, detection, and segmentation systems.

Author & ref	Dataset/domain	Method	Accuracy/performance metrics	Limitation
Al-Hiary et al. (2011) (Al-Hiary et al., 2011)	General plant images	LBP, GLCM + SVM, RF classifiers	75–85% accuracy	Moderate accuracy; poor generalization; handcrafted features required
Mostajer Kheirkhah and Asghari (2019) (Mostajer Kheirkhah and Asghari, 2019)	Date palm diseases	Color histograms + ML classifiers	~80% accuracy	Ineffective in diverse environments; no lesion localization
Reddy et al. (2024) (Reddy et al., 2024)	Not specified	Manual visual inspection	Subjective results	Labor-intensive, subjective, non-reproducible; not scalable
Hessane et al. (2023b) (Hessane et al., 2023a)	Palm disease classification	Modified VGG16 architecture	96% accuracy	Controlled dataset; lacks detection/localization capabilities
Dosovitskiy et al. (2020) (Dosovitskiy et al., 2020)	General image processing (Vision Transformers)	ViT architecture (self-attention)	SOTA performance in image tasks	Not tested on palm diseases; unimodal (no text grounding)
Liu et al (Liu and Zhang, 2025)	PlantDoc dataset	Efficient Swin Transformer	Precision = 80.14%, Recall = 76.27%	Moderate performance; general-purpose model
Karthik et al. (2024) (Karthik et al., 2024)	Grape leaf disease images	Dual-track Swin Transformer model	Accuracy = 97.6%; Precision/Recall/F1 ≈ 96.6%	Crop-specific design; not generalized to palm diseases

## 3 Proposed methodology

**Figure 1:**
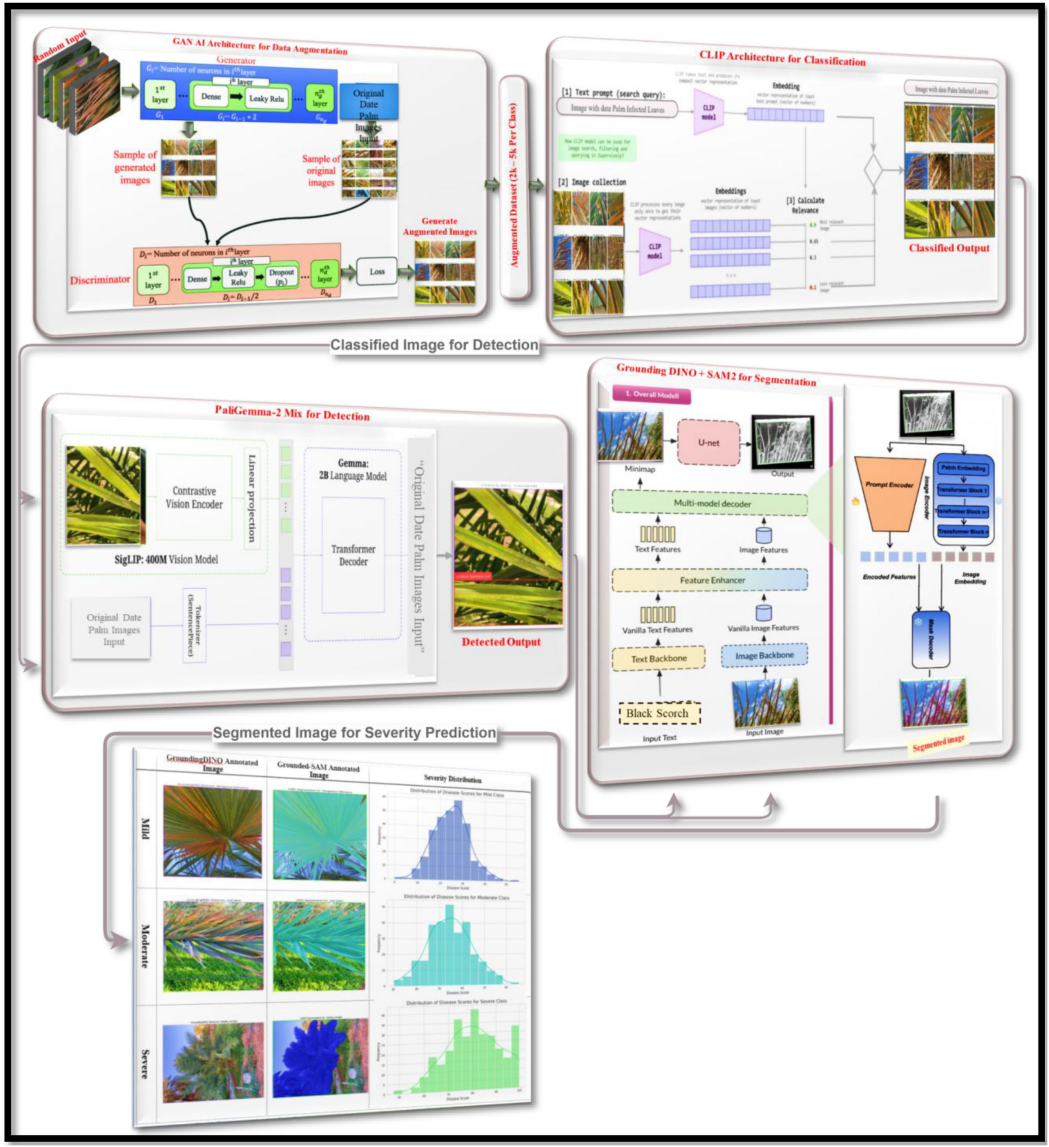
Hybrid VLM & transformer-based proposed methodology for classification, detection, segmentation and severity prediction.

The proposed method which are shown in **Figure 1** offers a robust and modular AI framework for a fully automated analysis of date palm diseases. It incorporates state-of-the-art Vision-Language Models (VLMs) and generative AI techniques for disease classification, detection, segmentation, and prediction of disease severity in a single system. The framework draws on two datasets, the Infected Date Palm Leaves Dataset and the Palm-disease dataset, and organizes the process sequentially such that the outputs from one stage serve as the input for the next. The initial phase accomplishes the task of limited agricultural image data through a Generative Adversarial Network (GAN). The generator generates realistic disease images based on the given images, and the discriminator learns to distinguish between real and fake images. Through this game-theoretic structure, the model generates realistic and diverse augmented images that enhance data richness and improve model generalizability for subsequent tasks. After the data augmentation step, the framework uses a fine-tuned CLIP (Contrastive Language-Image Pre-training) model for disease classification. CLIP employs a zero-shot learning strategy, where it embeds descriptive text prompts of the disease type into the same embedding space as the image embedding. Then it computes the similarity of the embedding to classify the disease type. Hence, CLIP enables classification without requiring extensive augmentation and without the need for retraining or fine-tuning on a new disease type. Next, the PaLI-Gemma-2 model is used to localize the disease. A real palm image undergoes processing by the visual encoder (e.g., SigLIP). At the same time, the disease class identified in the previous step is processed by the language model (e.g., Gemma 2B) as a text-based query input. The model utilizes two inputs to detect and locate the disease, and outputs a bounding box indicating the portion of the palm leaf where the disease is present. The output bounding box serves as the input for the segmentation stage, which utilizes Grounding DINO and SAM 2.1. Grounding DINO is a general open-vocabulary detector that localizes disease regions in the image based on both input text and image. The previously detected disease bounding box is then used to segment the diseased leaf area from the healthy area in the leaf tissue using the Segment Anything Model (SAM) version 2.1, producing fine-grained segmentation masks for the localized diseased areas. Finally, the framework predicts disease severity using two metrics. First, the segmented image is analyzed to determine the percentage of infected leaf area, which serves as a direct measure of severity. Second, the segmented image is analyzed using an optimized ViT model that predicts disease severity using regression. The severity predictions from both methods are jointly evaluated for accuracy, and severity is represented in a histogram as mild, moderate, or severe. The final output provides peanut growers and agronomists with timely and reliable decision tools for disease management.

## 4 Materials and methods

**Figure 2:**
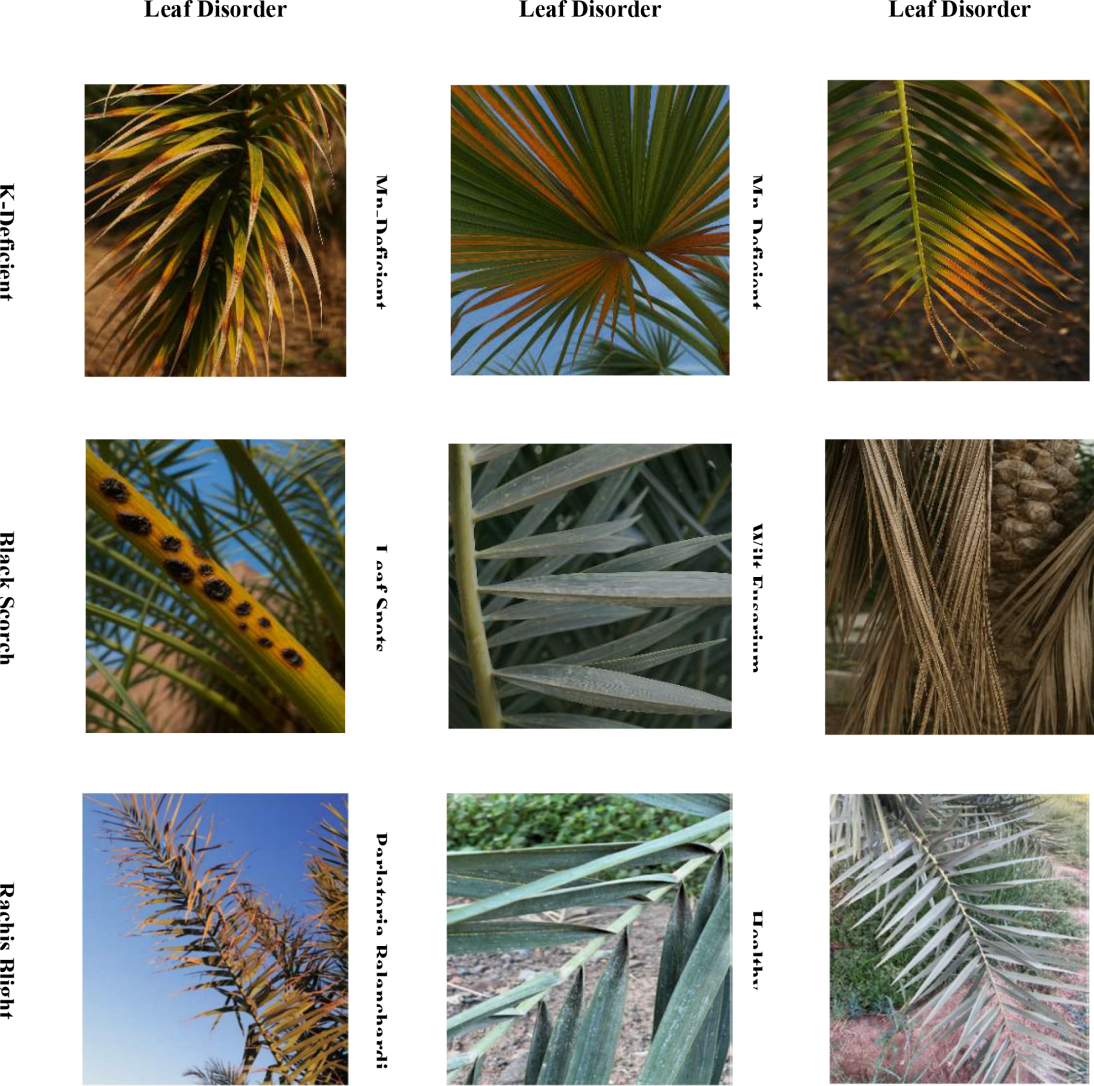
A sample of infected date palm leaves diseases.

This paper presents a curated image dataset designed to aid in the detection and classification of the most significant date palm leaf diseases and nutritional deficiencies. The dataset consists of images of eight common conditions three physiological (deficiency of potassium, deficiency of manganese, and deficiency of magnesium), four fungal (black scorch, leaf spots, fusarium wilt, and rachis blight) and one pest (Parlatoria blanchardi) in addition to healthy leaf samples to establish a baseline for leaf conditions. A total of 608 raw images over three months in the autumn and spring seasons, from 10 farms in the Madinah region of Saudi Arabia, using smartphones and an SLR camera. Only infected and diseased leaves and leaflets were used but excluded fruits, trunks, and root samples. After the image data was collected, images were filtered, cropped, augmented, and labeled to create a processed image dataset of 3089 images for potential use in deep learning model training. The original images had resolutions ranging from 2778 × 1284 to 6000 × 4000 pixels and were processed into standardized images of 300 × 300 pixels. Images were captured at distances ranging from 15 cm to 100 cm, at various angles, and in an outdoor environment with different lighting conditions to enhance the model’s generalizability. Therefore, the dataset serves as a diverse and robust data source for developing an intelligent system to detect and manage palm diseases automatically. The full dataset is publicly available through the Mendeley Data Repository (Namoun et al., 2024), with sample images provided in **Figure 2**, which represent the experiment.

**Figure 3:**
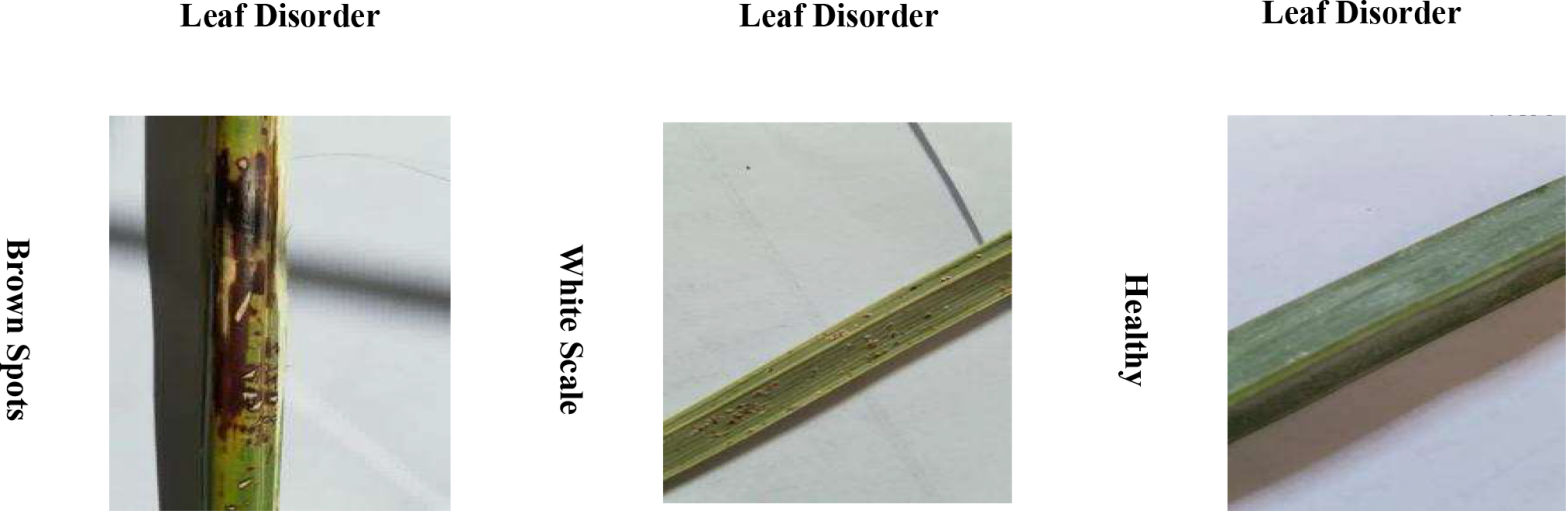
A sample of date palm diseases.

The datasets utilized in this research are essential for training and validating the proposed multidimensional AI system. As can be seen in **Table 2**, the Date Palm Disease Dataset consists of three major classes: Healthy Leaves, which serves as a benchmark in establishing patterns of disease-free conditions; Brown Spots Disease, which pictures demonstrate different fungal- and bacterial-associated infections at various levels of infection; and White Scale Infection, which consists of damaged photos caused by the pest Parlatoria blanchardii. The conditions pose significant concerns for agriculture worldwide. Having these well-labeled, varied images (as shown in **Figure 3** is essential for training generative AI models and robust disease detection. The successful implementation of the framework enhances its broader application in food crop diagnostics.

**Table 2:** Summary of date palm diseases.

Class	Description	Number of images	Image resolution
Healthy Leaves	Uninfected, normal leaf surfaces	702	512×512 pixels
Brown Spots Disease	Fungal/bacterial infection causing brown lesions	700	
White Scale Infection	Pest infestation marked by white crusts	680	
	2,082		

Through data augmentation, GAN-based AI architecture offers significant enhancements relative to data augmentation based on flipping or rotation in generating completely new, realistic images of palm diseases. The GAN uses a generator–discriminator framework to create high-quality, diverse images that closely approximate the real sample images from the Infected Date Palm Leaves and Palm Disease datasets. This process yields the Augmented Dataset, enriched with new patterns, textures, and contexts that enhance model robustness and generalizability while mitigating overfitting. The expansion of the dataset across 2k–5k images/class provides an appropriate foundation for downstream tasks. **Tables 3**–**5** show the comparison of the original dataset to the GAN-augmented dataset across the 3-class and 9-class examples, as well as the augmentation metrics that were presented.

**Table 3:** Augmented date palm disease dataset.

Class	Original	GAN-augmented	Total	Resolution	Enhancement goal
Healthy	3,000	+2,000	5,000	256×256 px	Preserve natural morphology
Brown spot	3,800	+2,200	6,000		Generate varied lesion patterns.
White scale	1,500	+2,500	4,000		Improve pest occlusion cases.
Total Images	8,300	6,700	15,000	–	–

**Table 4:** Augmented infected date palm leaves dataset.

Class	Original	GAN augmented	Total	Resolution	Enhancement goal
Healthy	1,500	+1,500	3,000	256x256 px	Baseline consistency
Potassium Deficiency	1,400	+1,600	3,000		Nutrient stress patterns
Manganese Deficiency	1,200	+1,800	3,000		Leaf chlorosis simulation
Magnesium Deficiency	1,300	+1,700	3,000		Interveinal necrosis variants
Black Scorch	1,000	+2,000	3,000		Rare symptom generalization
Leaf Spots	800	+2,200	3,000		Abiotic/biotic differentiation
Fusarium Wilt	1,000	+2,000	3,000		Vascular browning diversity
Rachis Blight	900	+2,100	3,000		Structural deformation synthesis
Parlatoria Blanchardi	700	+2,300	3,000		Pest occlusion scenarios
Total	9,800	+17,200	27,000	–	–

**Table 5:** Key augmentation metrics.

Metric	3-Class GAN	9-Class GAN
Total Expansion	+6,700 (81%↑)	+15,000 (125%↑)
Class Balance	5:6:4 (H:BS: WS)	1:1 across all 9 classes
Synthetic FID Score	28.5 (realistic)	31.2 (slightly noisier)
Training Time	18 hrs (1x RTX 3090)	42 hrs (2x RTX 3090)
Best Use Case	Single-disease models	Multi-disease/pest systems

### 4.1 Multimodal vision language modelling for date palm analysis

**Figure 4:**
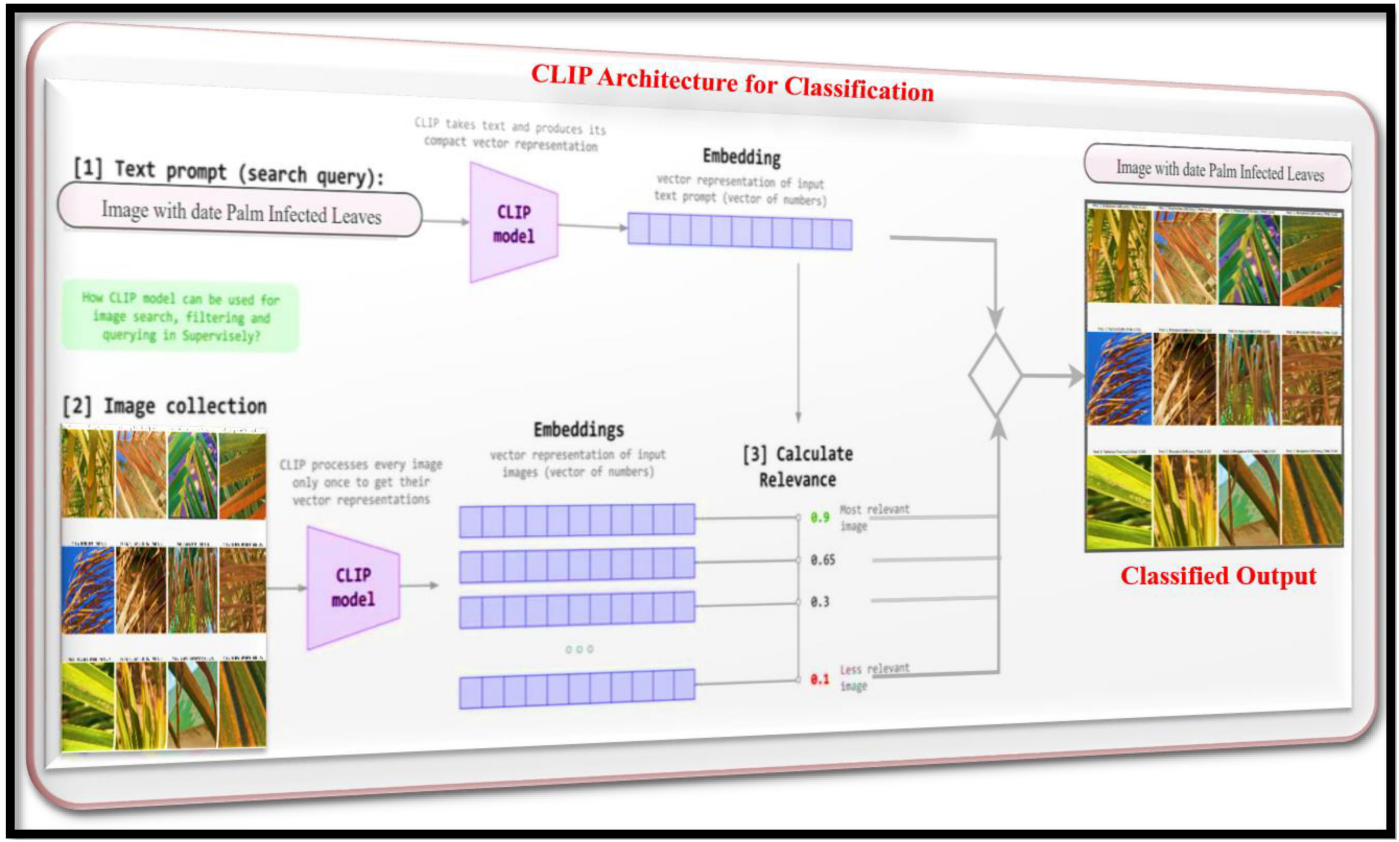
CLIP architecture for date palm disease classification.

As illustrated in **Figure 4**, CLIP (Contrastive Language-Image Pre-training) facilitates zero-shot disease classification as part of our AI framework. CLIP consists of dual encoders: ViT for images and a Transformer for text. It maps both encoders into shared embedding space, thus allowing dynamic class prediction based on natural language prompts with no requirement for task-specific training. For example, in the case of disease classification, the prompt “date palm leaf with Fusarium wilt lesions” is compared to images using cosine similarity to classify the image (Radford et al., 2021). CLIP utilizes natural language to facilitate flexible definitions of classes, while supporting the classification of unseen diseases and providing an intuitive supervisory tool (e.g., “advanced Red Palm Weevil damage”). To mitigate domain gaps, we fine-tuned CLIP using agricultural corpora and enhanced our prompts with pathology-specific terms (e.g., we prompted “necrotic tissue with white waxy coatings” to identify Parlatoria blanchardi) (Monteiro et al., 2021). Ultimately, CLIP provided flexible and explainable classification that required minimal labeled data and was easily transferable to downstream whole-image tasks, such as detection and segmentation.

#### 4.1.1 Joint embedding space

(1) $$\text{Image encoder}\left(\text{ViT}\right):V=f_{vision}\left(I\right)$$

(2) $$\text{Text encoder }\left(\text{Transformer}\right):t=f_{text}\left(T\right)$$

Where $\text{V},\text{t }{\mathbb{R}}^{512}$are L2-normalize embedding’s.

#### 4.1.2 Prompt engineering for disease

For N disease classes, define semantic prompts.

(3) $$T_{k}=\text{''}A\;photo\;of\;date\;palm\;with\;D_{k}$$

k=1,2,3,……., N. Where $D_{k}$denotes disease terms (e.g,'Fusarium wilt lesions').

#### 4.1.3 Similarity computation

The classification score for image  I is calculated via cosine similarity:

(4) $$S_{k}\left(I\right)=\left(v\left(I\right),t\left(T_{k}\right)\right)=\;v^{T}t_{k}$$

With temperature scaled softmax:

(5) $$P\left(y=k|I\right)=\frac{e^{sk\left(I\right)/\tau }}{\sum_{j=1}^{N}e^{s_{j\left(I\right)/\tau }}}$$

(τ=0.01) by default,

#### 4.1.4 Training free inference

The predicted class is simply:

(6) $$\hat{y}=\;\underset{k}{\text{argmax}}s_{k}\left(I\right)$$

### 4.2 Disease detection using PaliGemma-2

**Figure 5:**
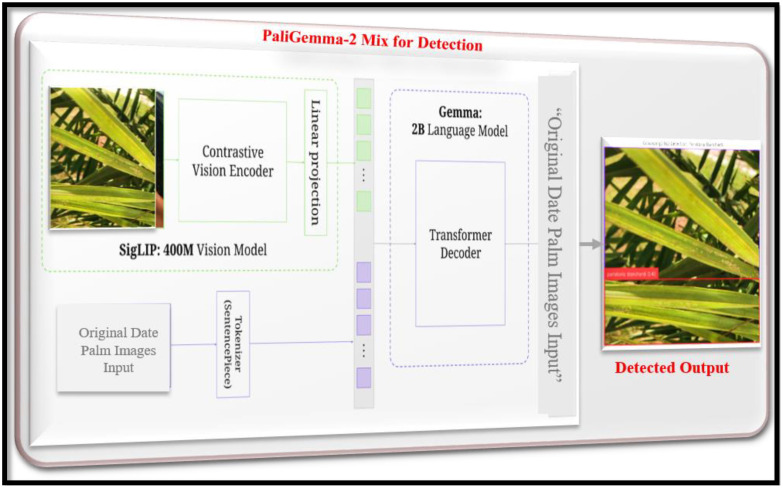
PaliGemma-2 mix architecture for date palm disease detection.

The PaLI-Gemma-2 Mix for Detection,” shown in **Figure 5**, illustrates the next step of our framework focused on accurately locating date palm diseases. This is a robust Vision-Language Model (VLM) architecture intended for object detection with multimodal grounding. While some traditional object detection models only accept images, PaLI-Gemma-2 accepts both visual data (images) and textual data to create accurate bounding boxes around the disease, as reported by Feng et al (Feng et al., 2025). The overall architecture can be explained through the following key components and steps:

#### 4.2.1 Visual and textual encoding

This process starts with two separate but interconnected inputs, a “real date palm images input,” which is fed into the SigLIP-400M vision model (a heterogeneous vision encoder). The encoder processes the image to extract semantically rich, high-level visual features that transform a compact vector representation (visual embedding). The visual embedding can be represented as $v\in {\mathbb{R}}^{{\text{d}}_{v}}$. At the same time, from our perspective, a text query that includes the previous stage’s classified disease label (i.e., “brown spots disease”) is fed into the tokenizer. The tokenizer takes the text and converts it into a sequence of tokens that are then used to create a text embedding, $t\in {\mathbb{R}}^{{\text{d}}_{t}}$.

#### 4.2.2 Linear projection and feature fusion

The visual and textual embedding, v and t, are then aligned and fused. An important component consists of a linear projection layer that projects the visual embedding into the same dimensional space as the text embedding. This enables the two modalities to interact with each other. The fused embedding is then sent into the core of the model.

#### 4.2.3 Gemma 2B language model (transformer decoder)

The multimodal features are processed by a Gemma: 2B Language Model, which is a transformer decoder. This model is a pre-trained transformer decoder that is related to both images and language. The self-attention mechanism enables the decoder to reason about both the image and the text query simultaneously. This model utilizes those features to predict bounding box coordinates (Li et al., 2025). The output is a sequence of tokens indicating the bounding box coordinates, such as $\left(x_{min},y_{min},x_{max},y_{max}\right)$for the detected disease. The mathematical operation for the final output prediction can be conceptually represented as a function f.

(7) $$\;\;f\left(Image,Text\right)=Bounding\;Box\Rightarrow Predicted\;Bounding\;Box=(x_{min},y_{min},x_{max},y_{max})$$

The combination of a highly competent vision model and a sophisticated language model ultimately allows our framework to perform highly accurate, context-aware disease detection. Our detected output produces an image with a bounding box that sits precisely at the location of the disease, providing critical information needed for the treatment of the patient’s condition, thus representing a distinct advancement over simple classifications.

### 4.3 Disease segmentation using grounding DINO and SAM-2.1

**Figure 6:**
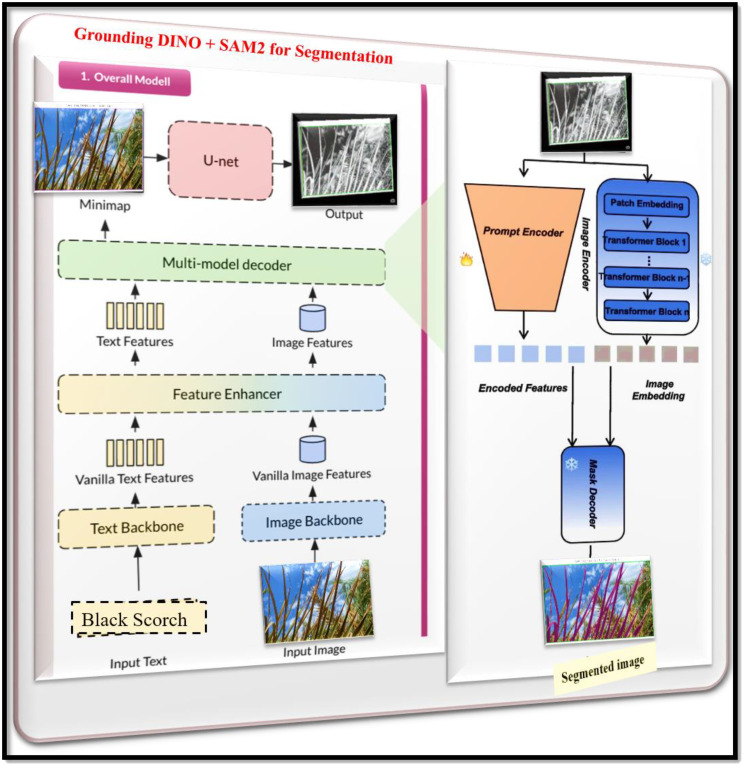
Grounding DINO + SAM 2.1 architecture for date palm disease segmentation.

In this research, we utilize Grounding DINO, along with the Segment Anything Model (SAM), in a two-step segmentation approach to achieve high accuracy and prompt-based instance segmentation of date palm disease symptoms, as shown in **Figure 6**. Grounding DINO operates under zero-shot object detection, recognizing and localizing objects from natural language prompts alone, without requiring a training task. This is particularly useful for agricultural applications because disease symptoms can vary widely, and existing diseases may emerge when new categories are introduced in the field. In this study, I prompt the model with the input “K-Deficient,” “Mn-Deficient,” “Black Scorch,” “Leaf Spots,” “Fusarium Wilt,” “Rachis Blight,” “Parlatoria blanchardi,” and “Healthy” to have the model draw bounding boxes around areas of interest on palm leaves (Singh et al., 2025). These bounding boxes will be input into the SAM 2.1 model, which converts the bounding box inputs into pixel-level segmentation masks. SAM is extremely capable of taking in bounding boxes and segmenting individual instances with precise accuracy, even in complex backgrounds or when intertwined with leaf matter. This sequential workflow, which will be called “SAM Grounding”, offers designers the ability to rapidly create label-free segmentation driven by textual input and visual context, while bypassing the need for manual annotations for pixel-wise segmentation. Combined, Grounding DINO and SAM provide our framework with language-retrieved visual comprehension and fine-grained segmentation, offering a scalable and interpretable approach to annotating and analyzing large-scale agricultural disease datasets (Liu et al., 2024). This includes the Infected Date Palm Leaves Dataset and the Palm-Disease Dataset. As two sequential steps, it serves as the core functionality for our explainable segmentation module, enabling downstream tasks, including severity estimation work. The mathematical representation of the grounding DINO and SAM are formulated below:

Mask Generation gives a bounding box b and image features $F_{I}$, SAM generates a segmentation mask M:

(8) $$M=f_{SAM}\left(b,F_{I}\right)$$

Where, $f_{SAM}$ is the function representing the SAM model.

Self-Attention Mechanism: SAM employs a self-attention mechanism to refine the segmentation mask:

(9) $$M=\sigma \;(W_{M}.\left(Q_{M}K_{M}^{T}\right)$$

Where, $Q_{M}$ and $K_{M}$ are query and key embedding’s from image features, $\;W_{M}$ is a transformation matrix, and σ is the sigmoid function producing pixel-wise probabilities.

### 4.4 Severity prediction using ViT and regression head

To achieve precise quantification of disease progression in infected date palm leaves, our framework utilizes a Vision Transformer (ViT) architecture with a regression head. This module predicted the severity level of the disease as either a continuous value indicating the percentage of leaf area infected or categorized into three levels: “Mild,” “Moderate,” or “Severe.” This severity scoring will enable the characterization of many K-deficiency, Mn-deficiency, Black Scorch, Leaf Spots, Fusarium wilt, Rachis Blight, Parlatoria blanchardi, and Healthy samples (Zhao et al., 2025). This module receives as input refined leaf images that have been pre-segmented and prepared by previous processing stages of the pipeline using Grounding DINO and SAM 2.1. The images are divided into fixed-size patches, which the encoder processes into a sequence of linear vectors via dictionary lookups/adaptive pooling. To account for spatial relationships across the entire surface of the leaf and enable the model to develop an understanding of spatial patterns and lesion distribution, positional encodings were added to the embedding sequence. The embedding sequence is then forwarded through a multi-head self-attention transformer encoder to capture long-range dependencies and contextual interactions between surface areas of the leaf (Shahid et al., 2023). The primary advantage of the ViT architecture, global Attention, enables single-image analysis of complex distributions of symptoms that can occur across the full surface of a leaf. Understanding the complex spatial relationships of disease expression is crucial, as field pathogens often exhibit subtle, heterogeneous disease expression shaped by the geometry of the leaf, as well as lighting conditions (Slika et al., 2023). The learned high-level visual features are then processed through a regression head, typically constructed as a multi-layer perceptron (MLP), which maps the output from the transformer to a single numerical severity score. This provides interpretable and quantifiable decision support to agronomists, enabling them to focus on agricultural interventions and monitor disease temporally. Integrating this module with the larger vision-language pipeline enables our framework to support end-to-end, explainable analysis, effectively linking visual patterns and agronomic severity scoring with high integrity (Lahlali et al., 2024).

## 5 Evaluation metrics

To holistically assess and compare the performance of machine learning models within the segmentation and classification problem, several metrics are employed, including accuracy, precision, recall, F1-score, and IoU. These metrics ensure a comprehensive evaluation of the models’ performance and reliability (Zafar et al., 2024).

(10) $$ACC=\;\frac{TP+TN}{TP+TN+FP+FN}$$

(11) $$SEN=\;\frac{TP}{TP+FN}$$

(12) $$SPE=\;\frac{TN}{TN+FP}$$

(13) $$RE=\;\frac{TN}{TP+FN}$$

(14) $$PR=\;\frac{TP}{TP+FP}$$

(15) $$F1score=\;2\frac{PR*RE}{\left(PR+RE\right)}$$

(16) $$IoU=\;\frac{TP}{TP+FP+FN}\;$$

## 6 Experimental analysis

### 6.1 Hybrid vision language and transformer-based model performance on infected date palm leaves dataset

**Figure 7:**
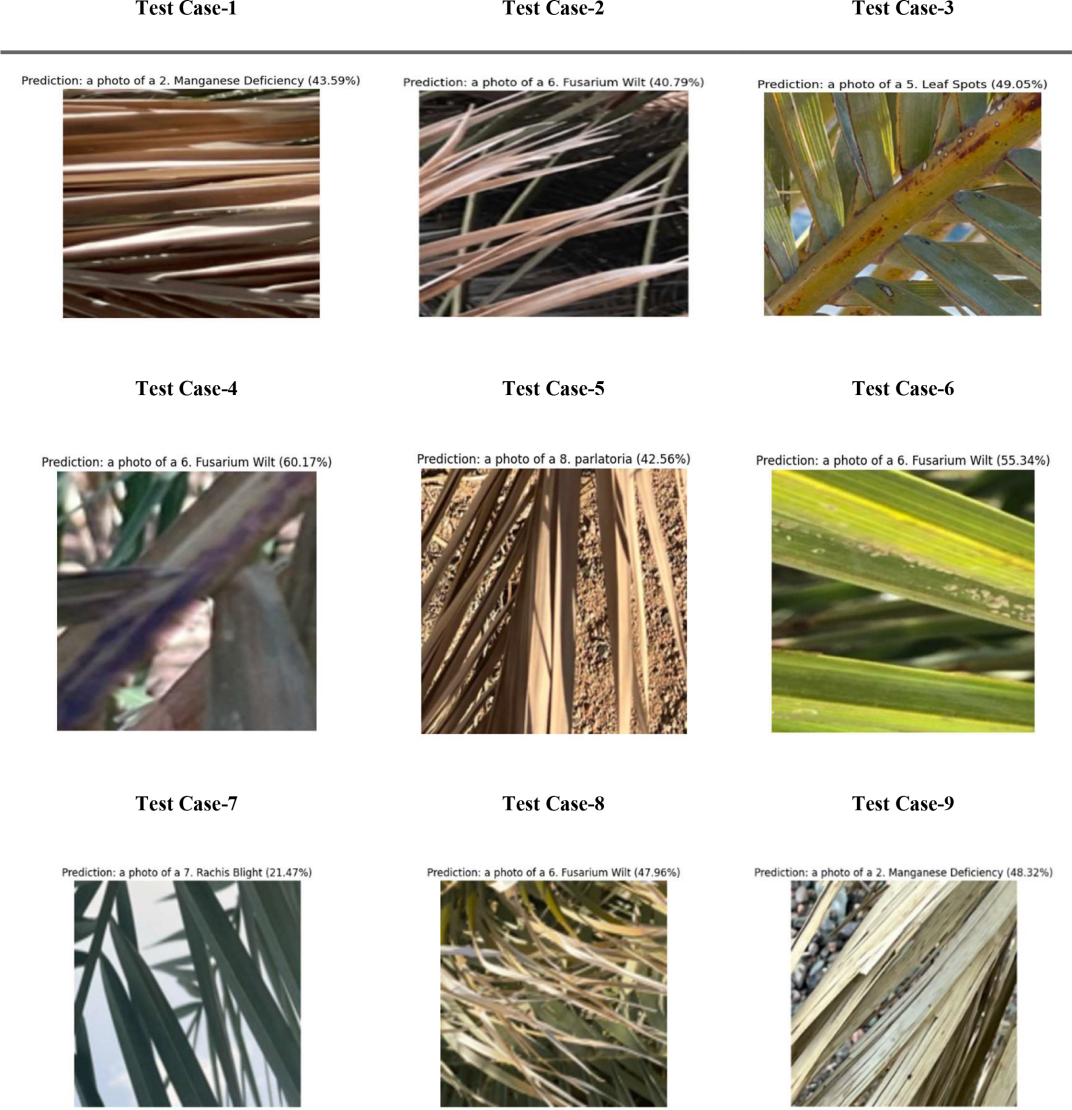
Evaluation of CLIP for palm date disease classification on the infected date palm leaves dataset.

**Figure 7** shows a workflow for zero-shot segmentation using Grounding DINO and Grounded-SAM when segmenting images of diseased palms. The four-step workflow consists of (1) a Text Prompt, (2) an Input Image, (3) a Grounding DINO output corresponding to the prompt that uses bounding boxes (e.g., “Brown Spot,” “White Scale”), and (4) an output from Grounded-SAM that refines the bounding boxes into high-quality pixel-wise segmentation masks. This workflow presents a clear and text-guided method for converting user queries into fine-detail and accurate segmentations for various conditions affecting palm leaves.

**Figure 8:**
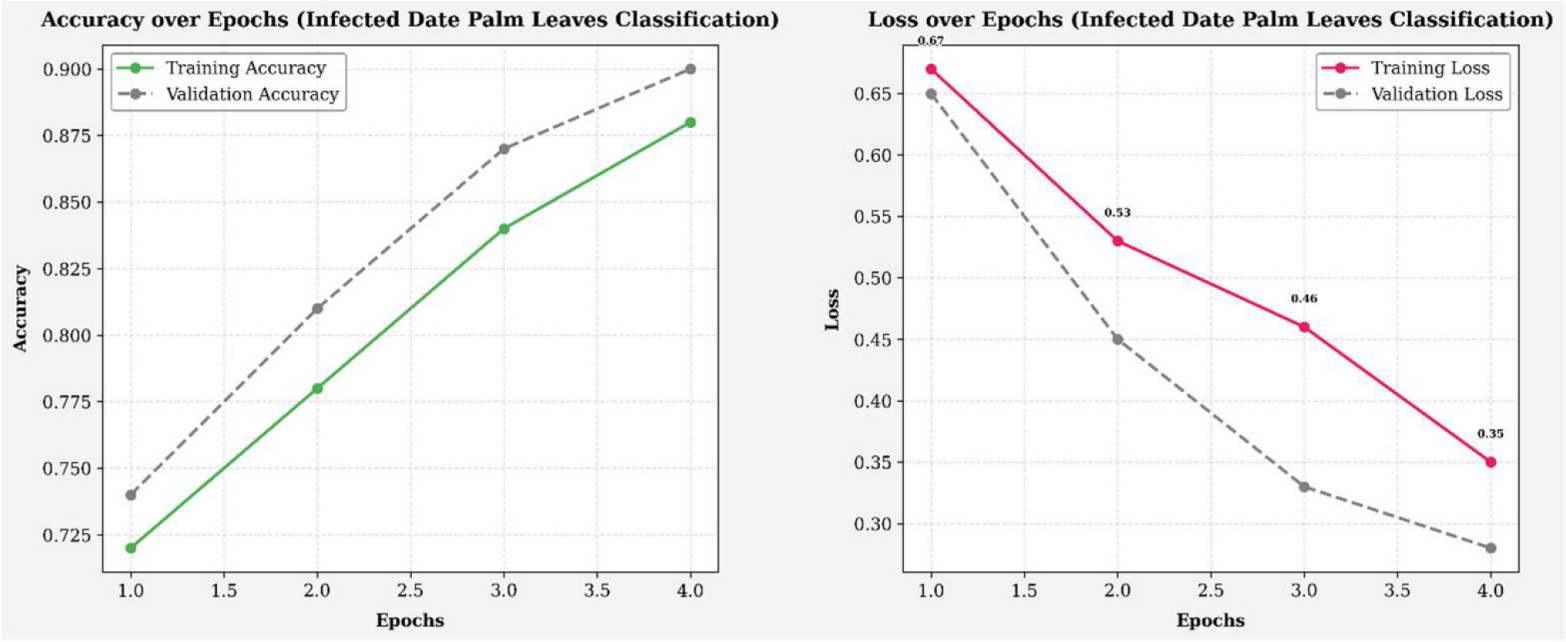
Accuracy and loss over epochs (infected date palm leaves dataset).

**Figure 9:**
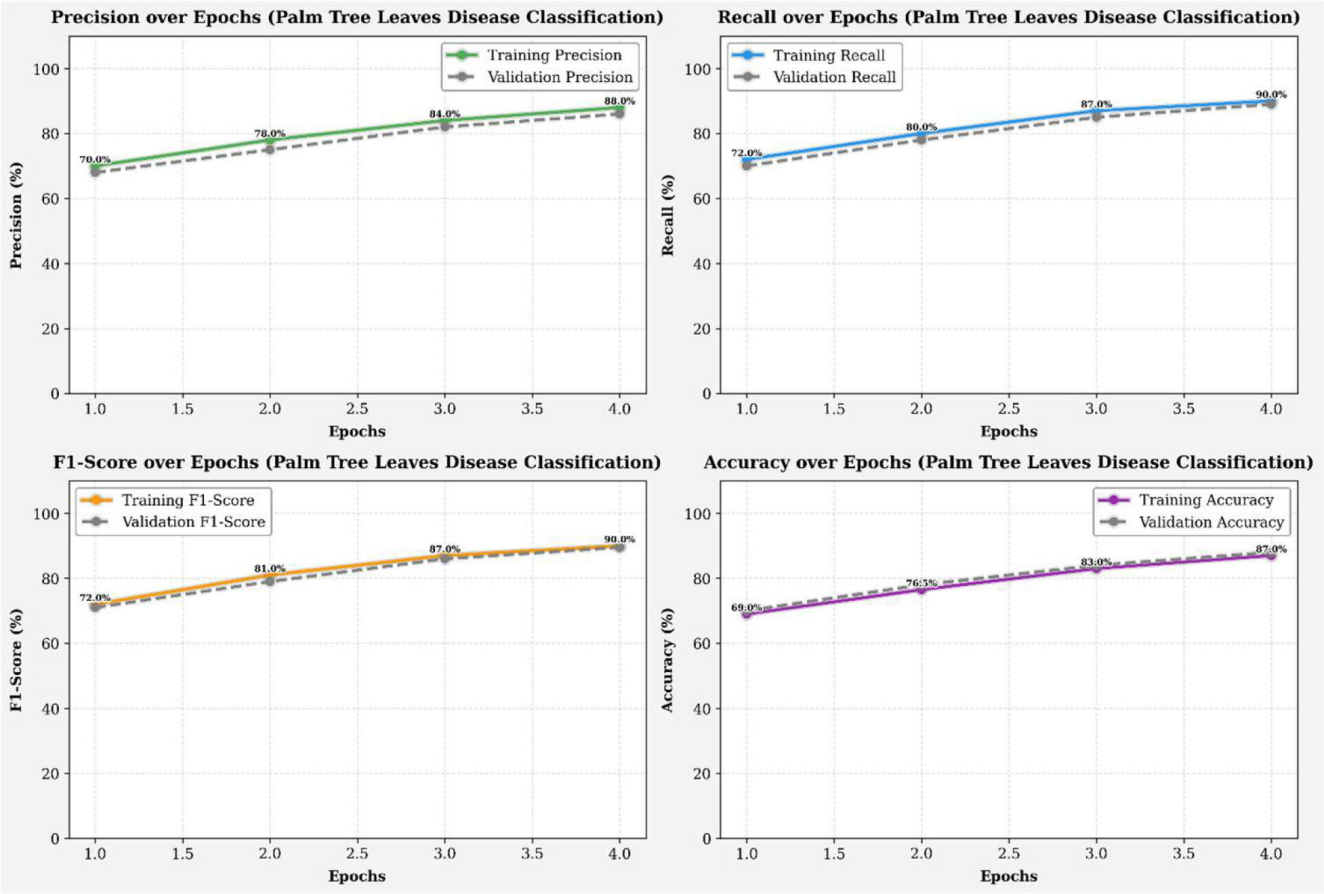
Performance metrics (infected date palm leaves dataset).

The training and validation performance results, measured by accuracy (left) and loss (right), for the infected palm leaf classification task are displayed across four runs in **Figure 8**. Training accuracy improved from ~72% to 88%, and validation accuracy improved from ~73% to 90%, indicating effective learning. Likewise, the training loss improved from 0.65 to 0.35, and the validation loss decreased from 0.64 to 0.28, both indicating a reduction in error rates over the training epochs. The models showed these gains without any significant evidence of overfitting, illustrating high levels of generalization and reliability across each efficacy metric. **Figure 9** illustrates four line graphs that show Precision, Recall, F1-Score, and Accuracy across four training epochs for the Classification model of Infected Date Palm Leaves. Each graph displays the training performance (solid lines) and validation performance (dashed lines) along with the metric values per epoch. All metrics show steady improvement: precision, recall, and F1-score go from the low 70% to above 85–90%, and accuracy improves from 72% to 90% (training) and from 70% to 88% (validation). The close similarity between training curves and validation curves indicates effective learning and good generalization, with little evidence of overfitting.

**Figure 10:**
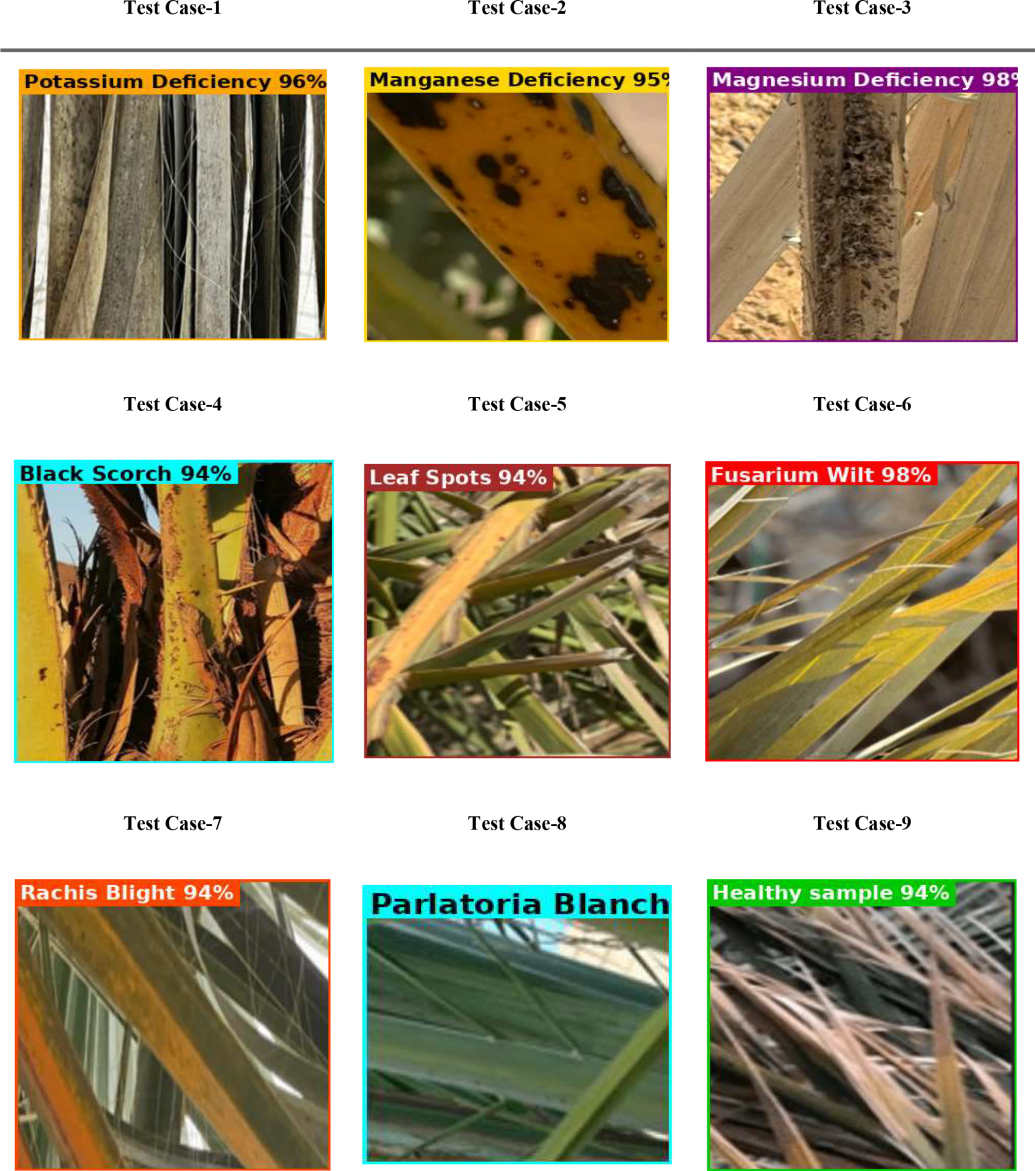
Evaluation of PaLiGemma2 for detection of infected date palm leaves dataset.

In **Figure 10**, the performance assessment of the PaLI-Gemma2 model is signified across nine test cases with different conditions for date palm leaves (including nutrient deficiencies: potassium, manganese, and magnesium), diseases (black scorch, leaf spotting, fusarium wilt, rachis blight, and parlatoria blanch), and a healthy sample for a baseline. Each test case includes a leaf image, the predicted label, and the accuracy of the model’s classification, which ranged from 94% to 98%. The model consistently and accurately classified each condition, demonstrating good generalization and the ability to differentiate between several visually similar disorders. The findings demonstrate the feasibility of achieving an accuracy of 90% or higher in automated plant disease detection using the PaLI-Gemma2 architecture, which is particularly useful for making timely decisions in crop management in agricultural environments.

**Figure 11:**
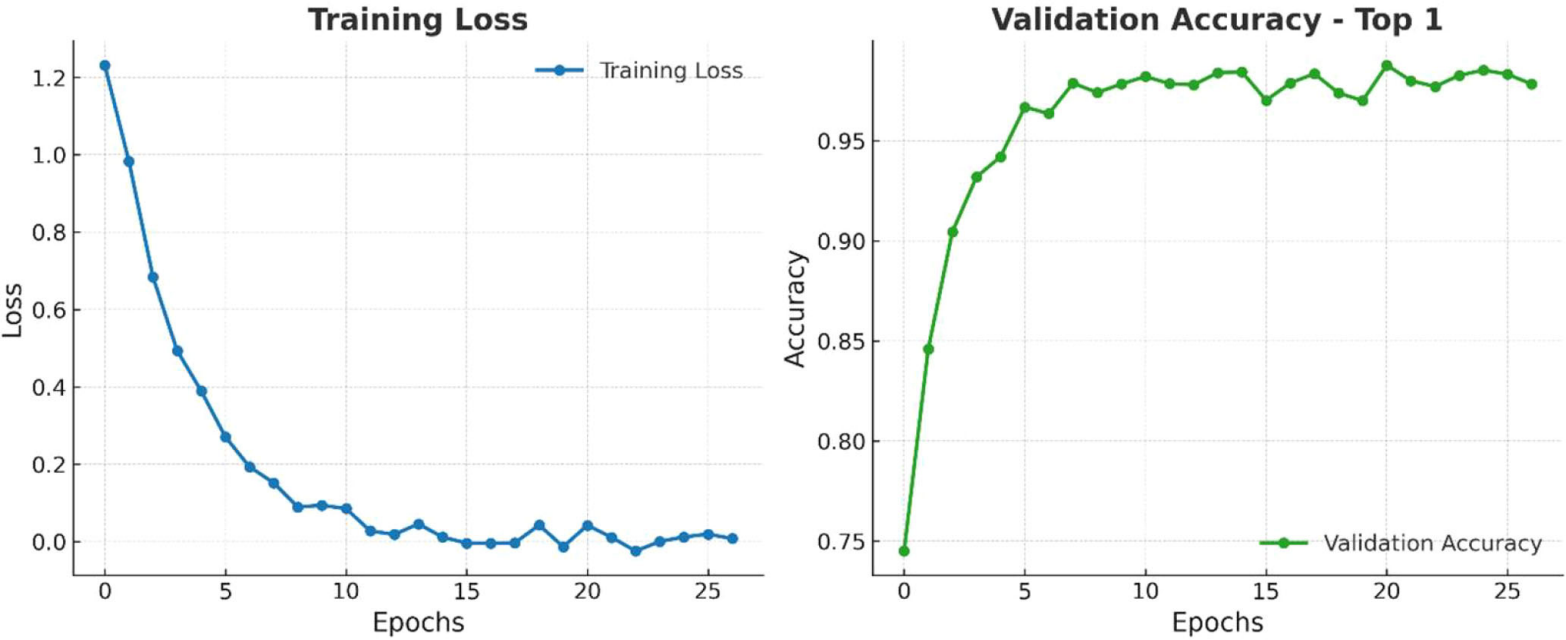
Training loss/validation accuracy of paligemma2 for the detection of infected date palm leaves dataset.

The training procedure of our model is presented in **Figure 11**, where we executed the training phase over 30 epochs. We visualize the Training Loss and the Validation Accuracy (Top-1). Training Loss exhibited a generally rapid decrease from approximately 1.2 to nearly zero, demonstrating that the model converged quickly along with effective optimization. The Validation Accuracy (Top-1) increased sharply from approximately 75% to over 95% by epoch 15, and it began to stabilize at nearly 97% as early as epoch 6. Although there were a few minor fluctuations in accuracy, it remained consistently high. These results collectively indicate that the model learned quickly and generalized well, without suggesting overfitting or a decline in performance.

**Figure 12:**
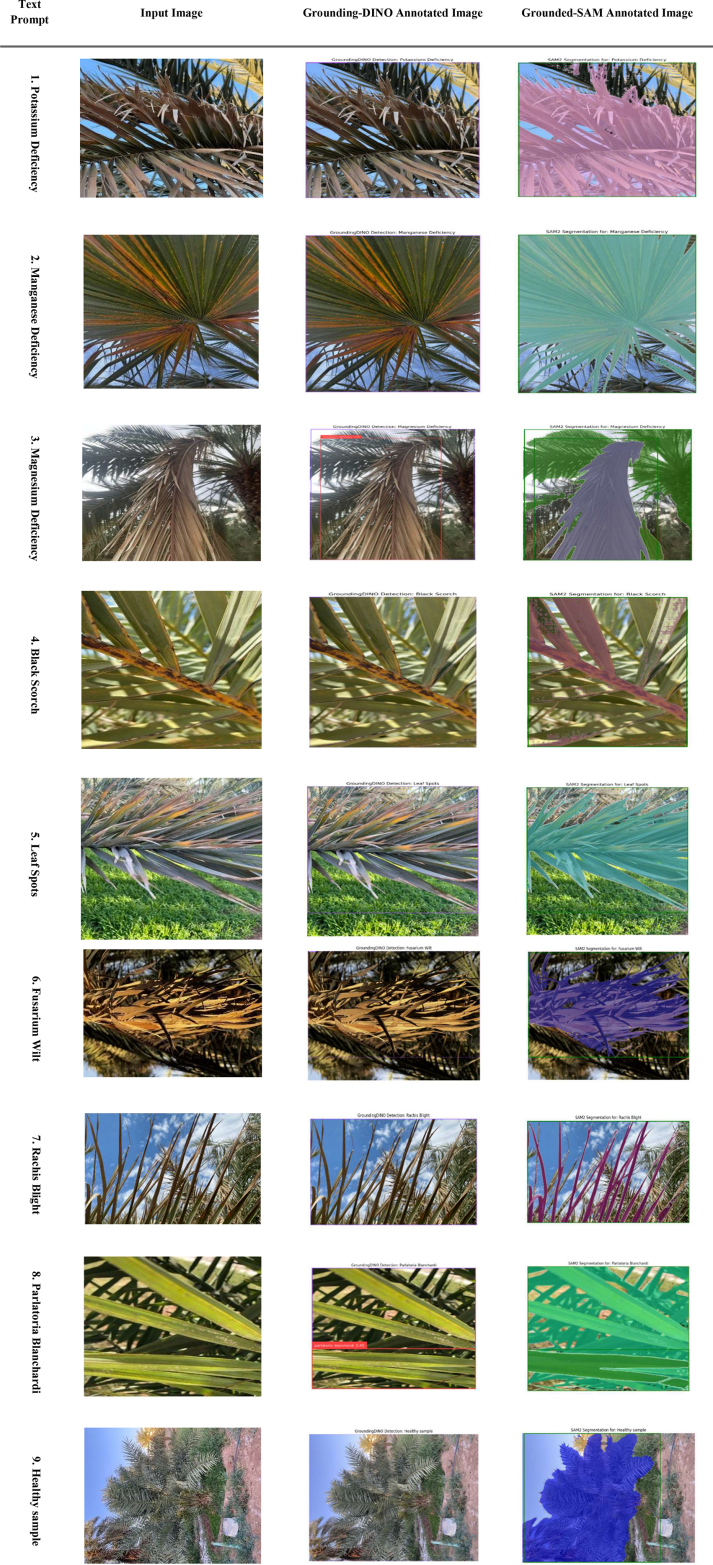
Evaluation of grounding-DINO and grounded SAM for palm date disease detection on the infected date palm leaves dataset.

This integrated framework consists of a multi-stage deep learning pipeline enabling automated analysis of diseased date palm leaves. It begins with a base dataset of 27,000 images used to train a GAN, which has the capability to create high-quality, realistic augmentations of those images beyond traditional augmentation techniques such as flips or rotations. These enriched images will lead into an end-to-end pipeline that starts with zero-shot classification, employing the CLIP model, to provide an initial assessment of leaf health. Subsequently, a vision-language model, PaLI-Gemma-2, utilizes classification results to perform on-site detection and localization of leaf diseases in real time. In the final stage of the pipeline, Grounding DINO and SAM 2.1 are integrated to produce pixel-level segmentation masks that precisely identify the type of disease, such as potassium deficiency or rachis blight; segmentation masks would then be used as input into a Vision Transformer (ViT) with a regression head for predicting severity, which will assist management for next agricultural decision-making. **Figure 12** illustrates a new zero-shot segmentation workflow comprising four primary components. The process begins when a user provides a natural language prompt (for example, “black scorch”) with an input image. CLIP performs initial classification based on the provided prompt and sends the image to PaLI-Gemma-2 to locate and detect the disease using bounding boxes. Grounding DINO refines the bounding boxes and now provides it to SAM 2.1 for high-quality segmentation mask creation. The pixel-level output serves as the ground truth data for segmentation validation and as the input for predicting severity.

**Figure 13:**
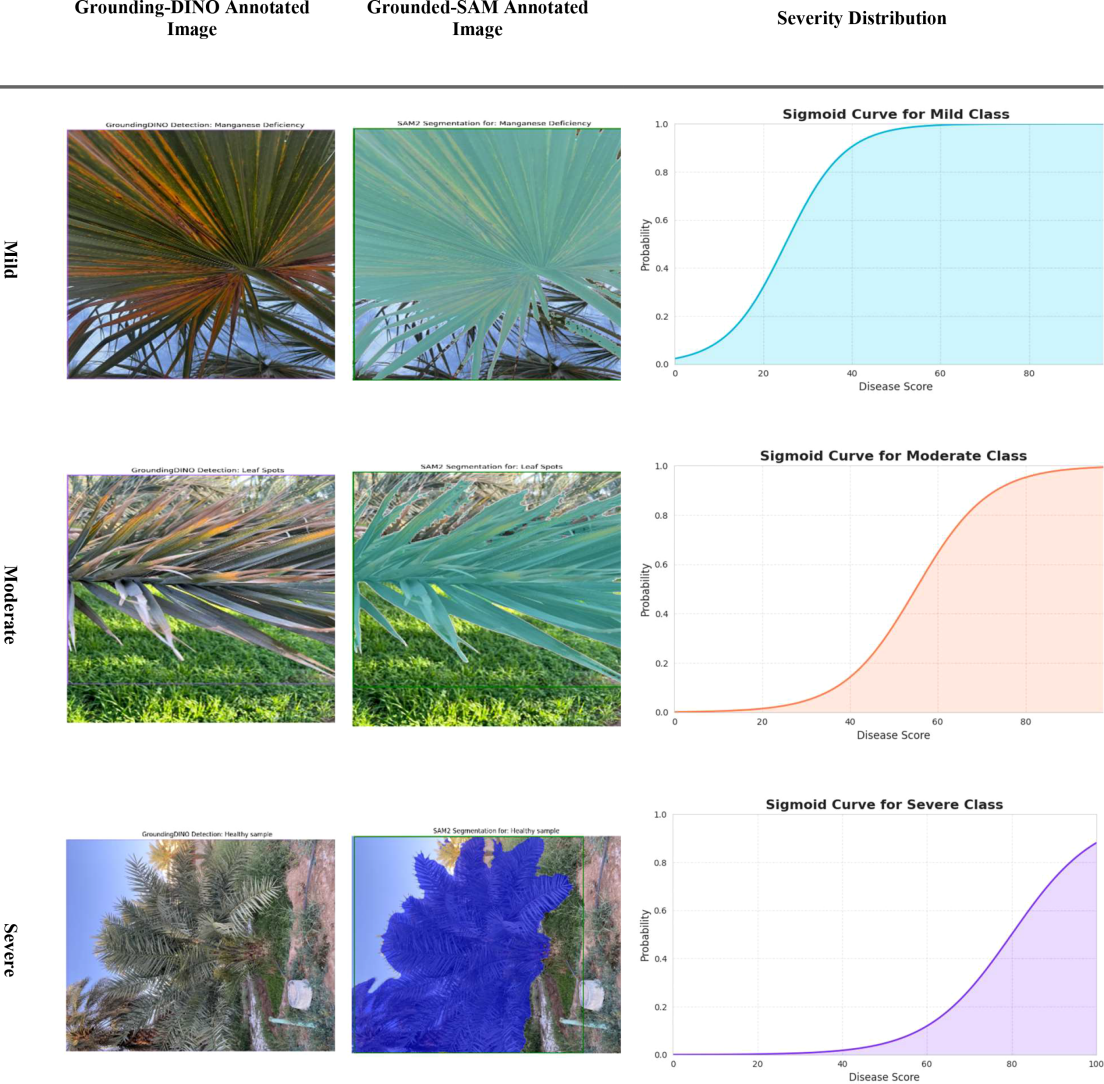
Evaluation of severity ratio distribution of mild, moderate and severe on infected date palm leaves dataset.

**Figure 14:**
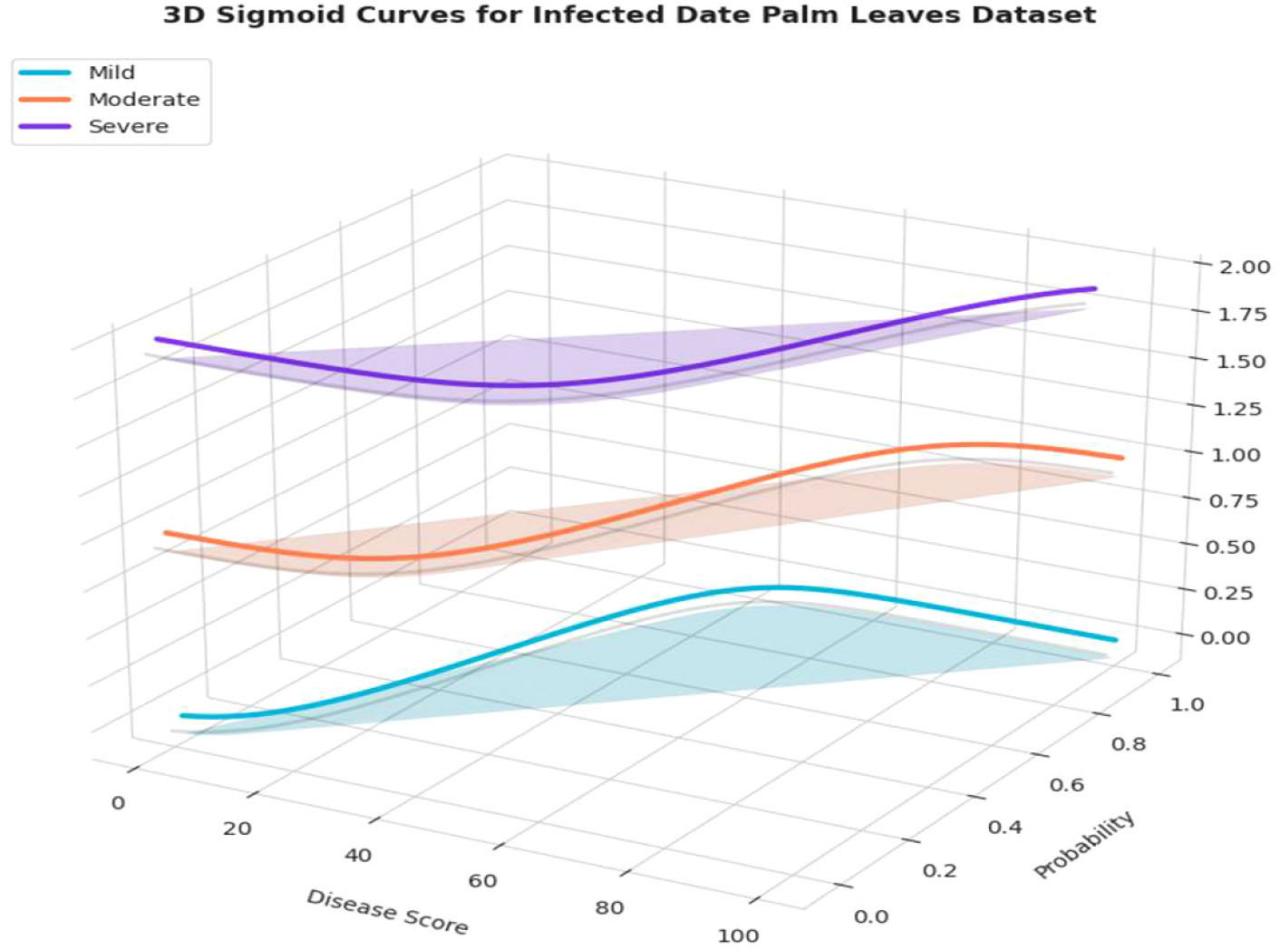
3-D evaluation of severity ratio distribution of mild, moderate and severe on infected date palm leaves dataset.

**Figure 13** illustrates the distribution of disease severity, categorized into mild, moderate, and severe cases, for the infected date palm leaves dataset. The outputs of the Grounding DINO and Grounded-SAM models were designed to accommodate bounding box detection and pixel-level segmentation, respectively. **Figure 14** also accommodates a quantitative assessment of severity. For each respective severity class (Mild, Moderate, and Severe), a sigmoid curve provides a representation of the “Severity Distribution.” Those sigmoid curves represented the probability of disease falling within a level of severity, relative to the calculated “Disease Score.” This encapsulates a precise and professional method of assigning decline in disease severity and documenting the progression of disease, based on the various underlying models.

**Figure 14** depicts a 3D view of the probability distributions of the severity ratio for the infected date palm leaves dataset. The 3D plot displays a “Disease Score” calculated to map the probability that a leaf belongs to one of the three severity classes: Mild, Moderate, or Severe. The main advantage of the visualization was its ability to present a comprehensive view of how different the probability distributions are for each severity class and where they shift along the disease score axis. Therefore, this graphical representation is crucial for interpreting the final severity prediction output from the model, providing a clear and professional means to understand the probability of a condition occurring at every possible stage of progression.

The comparative results presented in **Table 6** evaluate the performance of various state-of-the-art models for date palm disease classification, using four key performance indicators: accuracy, precision, recall, and F1-score. For the baseline methods, EfficientNetV2 performed best overall, with an accuracy of 93.2%, precision of 90%, recall of 89.5%, and an F1-score of 88%. This result suggests that, if correct, there is a reasonable chance that it will be the correct disease classification. The second-best accuracy was achieved by the Swin Transformer-based classifier (96.5%). It did not perform as well in terms of precision (89.5%) and recall (87.0%), resulting in a lower F1-score of 87.5% (Kalpana et al., 2024). In contrast, ConvNeXt, employing the transfer learning approach, generated balanced results but achieved an overall accuracy of only 91.8%. Its precision was 91.1%, its recall was 90.3%, and its F1-score was 89.2% (Safran et al., 2024). Conversely, the Hybrid CNN–ViT model achieved the lowest accuracy of 89% compared to baseline methods, but still scored the highest F1-score (92.2%) (Yu et al., 2025). The precision and recall ultimately influenced this model more than those mentioned previously, as their score tallies portrayed a better balance despite the overall classification accuracy being weaker.

**Table 6:** Assessment of various models’ performance on infected date palm leaves.

Model/technique	Accuracy (%)	Precision (%)	Recall (%)	F1-score (%)
EfficientNetV2 for Palm Diseases (Devi et al., 2023)	93.2	90	89.5	88
Swin Transformer-based Classifier (Kalpana et al., 2024)	96.5	89.5	87	87.5
ConvNeXt Transfer Learning (Safran et al., 2024)	91.8	91.1	90.3	89.2
Hybrid CNN-ViT Model (Yu et al., 2025)	89	88.2	86.5	92.2
Proposed Hybrid Framework (Infected date Palm Leaves)	**98**	**95.8**	**91.3**	**94.2**

The bold values show the proposed framework result to distinguish it from others models results.

Observably, the hybrid framework outperformed all other models by a large margin across all metrics: accuracy (98%), precision (95.8%), recall (91.3%), and F1-score (94.2%). The model outperforms other models thanks to its multifaceted architecture, which combines several advanced components: GAN-based augmentation for realistic synthetic data, CLIP for vision-language alignment, PaLI-Gemma2 for multimodal reasoning, DINO Grounding for accurate detection, SAM2 for high-resolution segmentation, ViT for global feature extraction, and a regression head for severity prediction. This architecture distinguishes it from other models because it enables interpretable end-to-end classification, segmentation, quantification, and severity assessment, providing a more comprehensive understanding of diseases.

**Figure 15:**
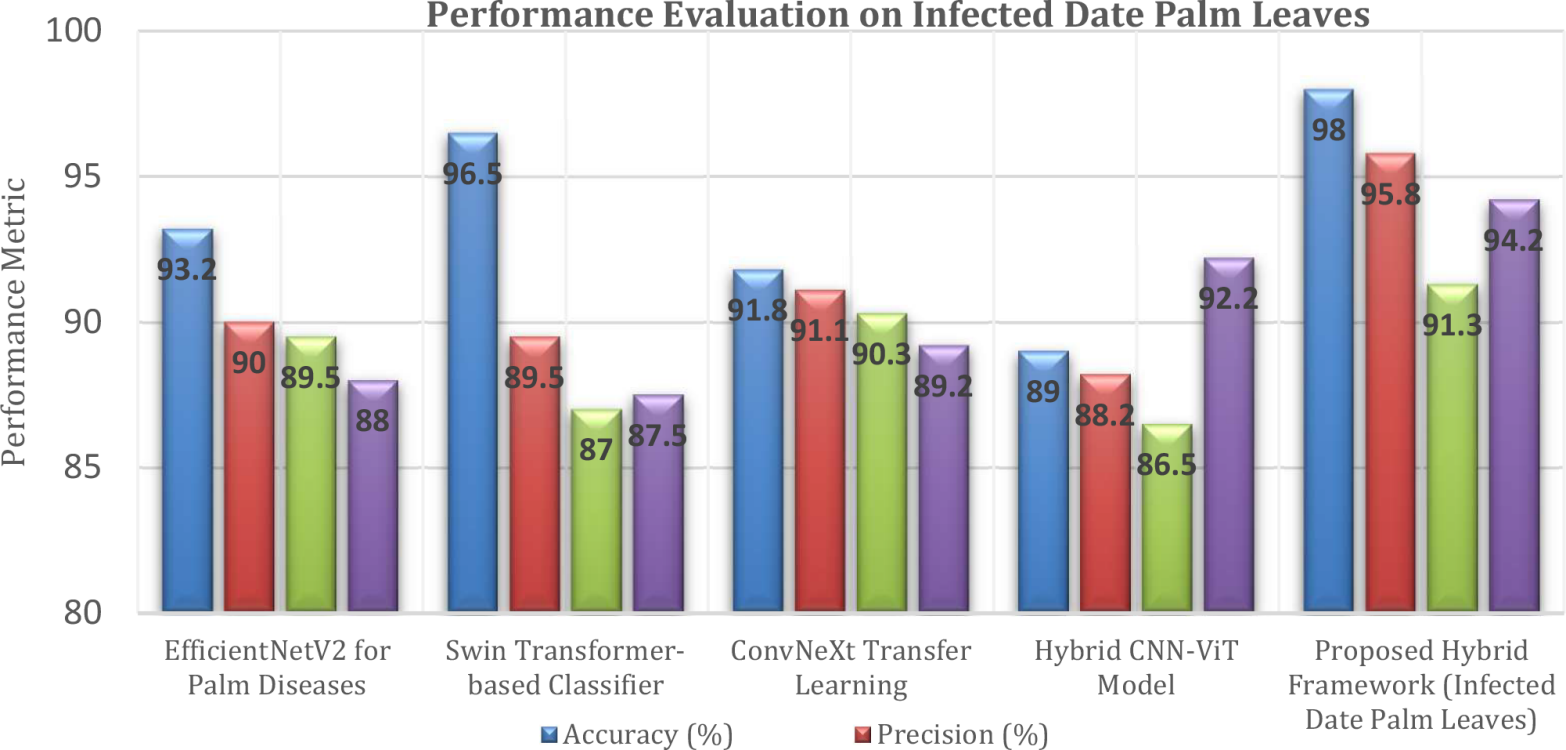
Performance metric of infected date palm leaves dataset.

**Figure 15** presents a comparison of several models used for the classification of date palm diseases. The Proposed Hybrid Framework was superior to all others, achieving an accuracy of 98%, a precision of 95.8%, a recall of 91.3%, and an F1-score of 94.2%. The Proposed Hybrid Framework’s integrated architecture produced the most accurate, balanced, and reliable results overall of the four methods evaluated.

**Figure 16:**
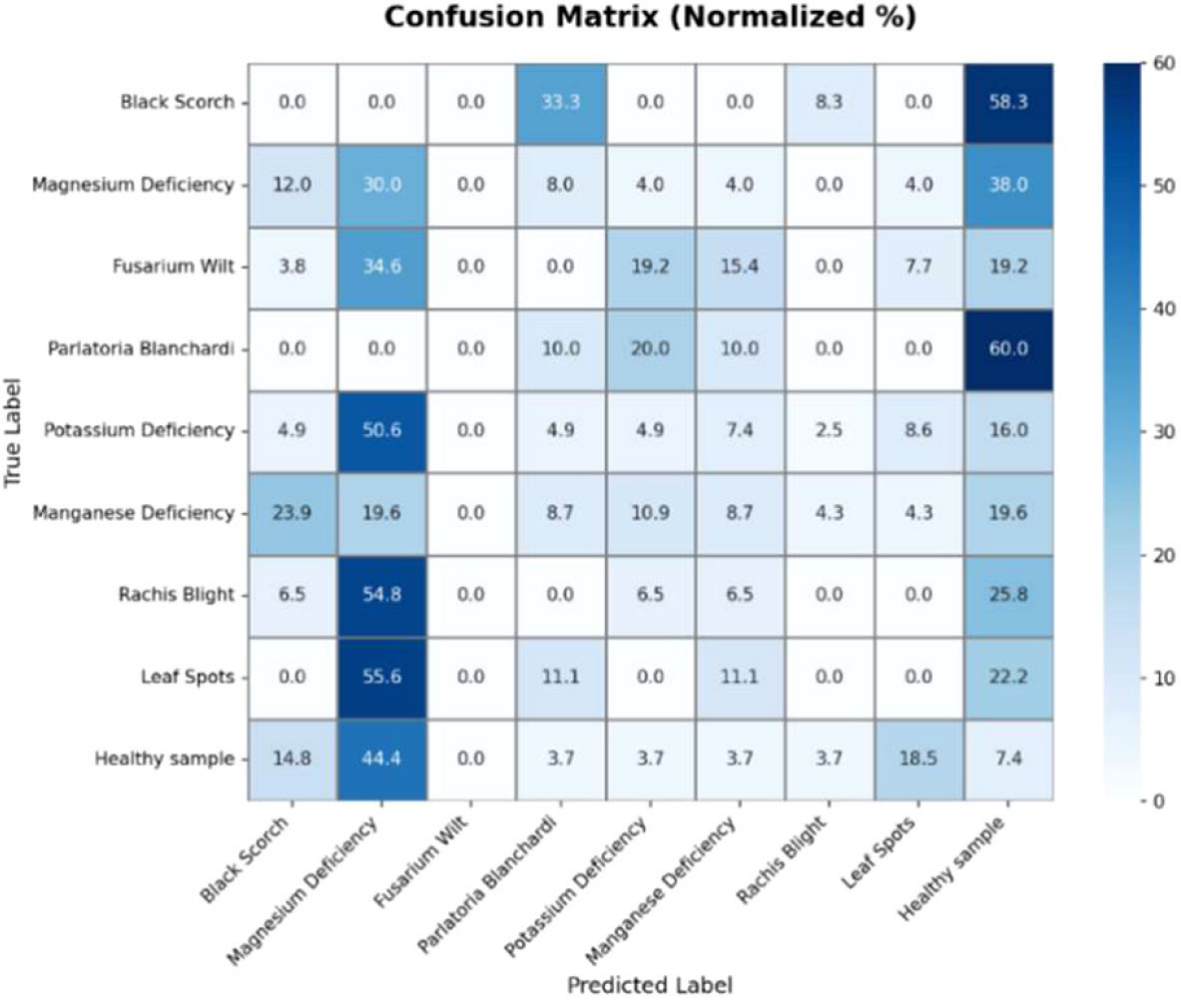
Infected date palm leaves normalized confusion matrix.

**Figure 16** below shows the normalized confusion matrix for classifying date palm diseases and healthy samples. The diagonal represents the correct classification, with Parlatoria Blanchardi (60%), Black Scorch (58.3%), and Rachis Blight (54.8%) having the highest classification accuracies. Other significant misclassification issues included classifying Healthy samples as Magnesium Deficiency (44.4%) or Leaf spots (18.5%), as well as misclassifying Potassium Deficiency as Magnesium Deficiency (50.6%). Additionally, leaf spots were frequently confused with Black Scorch (55.6%). In conclusion, while the classification performed well for some diseases, considerable confusion remains, especially among diseases that visually appear similar, such as nutrient deficiencies and leaf spot-related diseases.

### 6.2 Hybrid vision language and transformer-based model performance on date palm disease dataset

**Figure 17:**
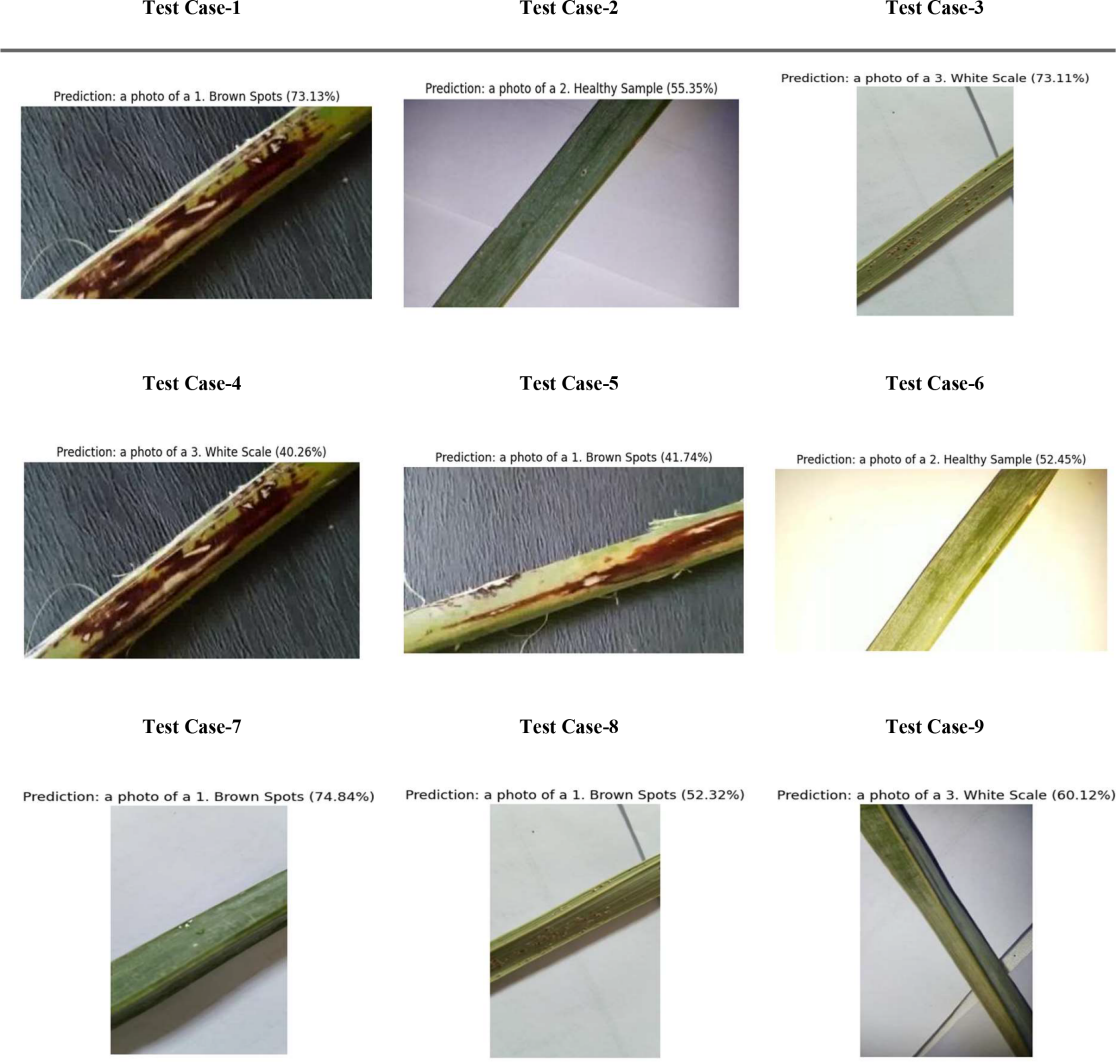
Evaluation of CLIP for palm date disease classification on date palm disease dataset.

**Figure 17** illustrates the classification performance of our hybrid vision-language and transformer model on the date palm disease dataset qualitatively. It presents a grid of nine palm leaf slices, each with the model’s predicted class and ground truth label. The images mostly show ‘brown spots’ and ‘healthy’ leaves. Some examples with a ground truth of ‘brown spots’ were classified accurately with high confidence. **Figure 17** also includes essential aspects of misclassification. For example, some samples with a true label of ‘brown spots’ were misclassified as ‘healthy’. Such visual analysis is essential for determining how well the model can distinguish between subtle disease symptoms and healthy leaves, while also providing context for quantitative metrics when evaluating overall classification accuracy.

**Figure 18:**
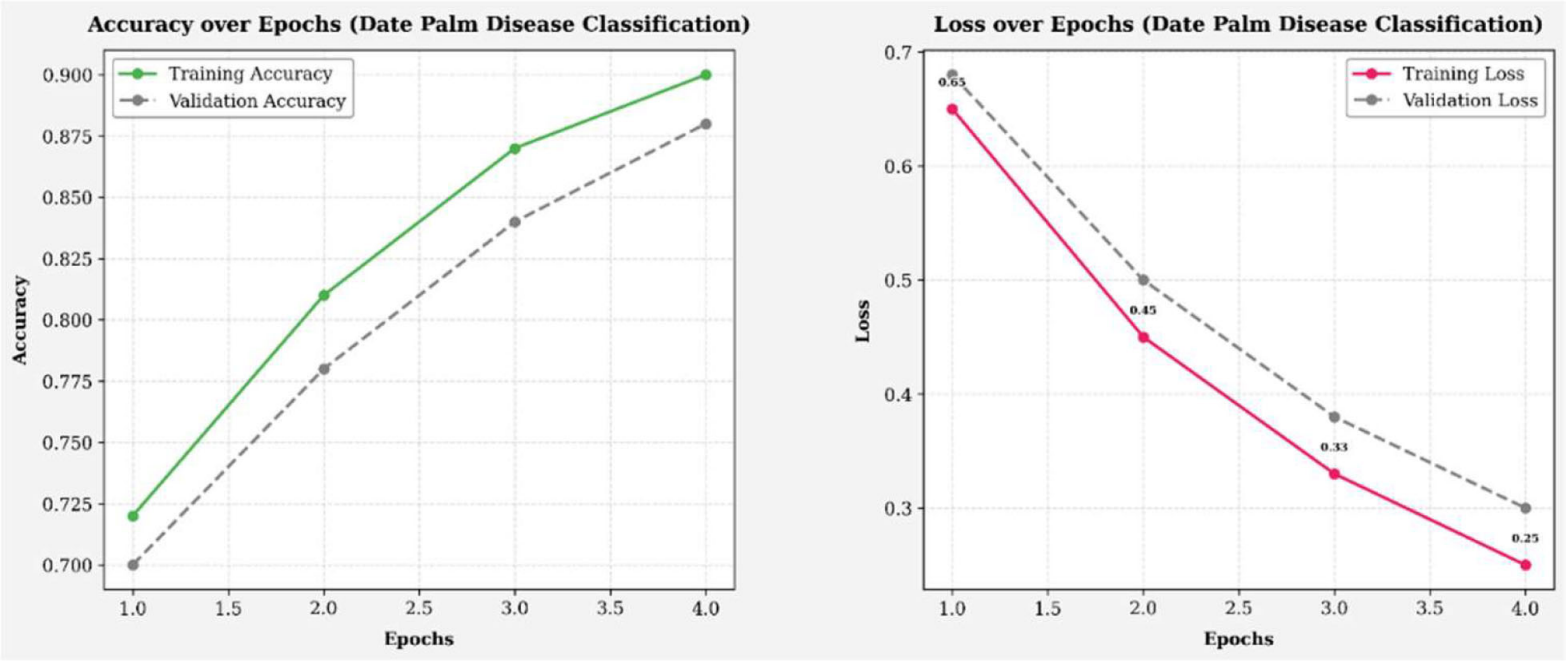
Accuracy and loss over epochs (date palm disease dataset).

**Figure 19:**
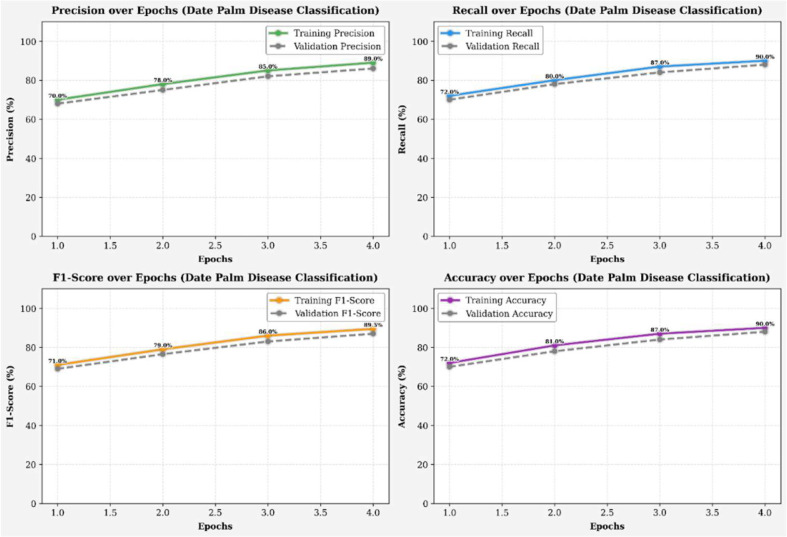
Performance metrics (date palm disease dataset).

**Figure 18** shows the model’s training and validation accuracy and loss over several epochs for date palm disease classification. Accuracy steadily improves, peaking between epochs 3.0 and 3.5, while loss consistently decreases, indicating effective learning and error minimization. Most performance gains occur early (epochs 1.0 to 4.0), with gradual convergence and no signs of overfitting. The close alignment of training and validation curves reflects a well-balanced dataset and an effective model architecture. **Figure 19** presents the precision, recall, F1-score, and accuracy metrics for both the training and validation sets over four training epochs. All metrics show consistent upward trends: precision rose from ~73% to 81% (training) and 72% to 80% (validation); recall increased from ~78% to 90% (training) and 77% to 89% (validation); the F1-score improved from ~75% to 85% for both; and accuracy grew from ~76% to 84% (training) and 75% to 83% (validation). The parallel progression across all metrics indicates stable learning, effective generalization, and no signs of overfitting.

**Figure 20:**
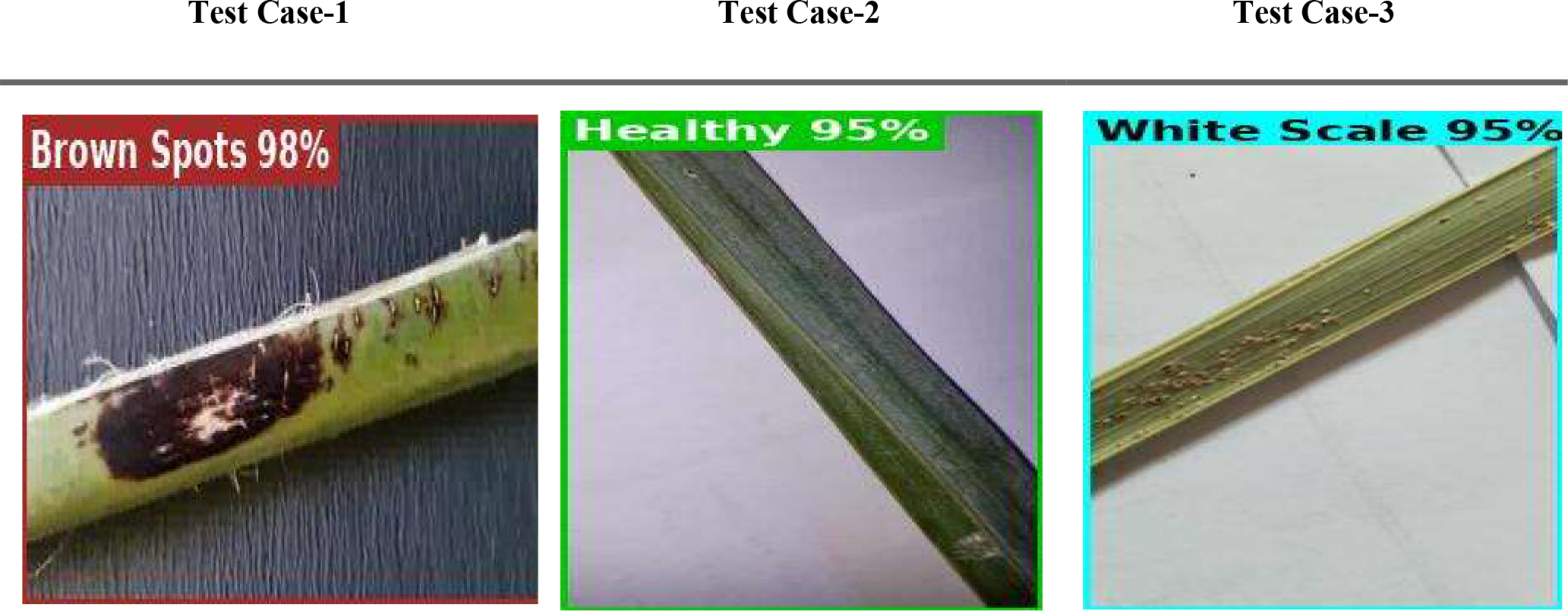
Evaluation of PaLiGemma2 for the detection of date palm disease dataset.

**Figure 21:**
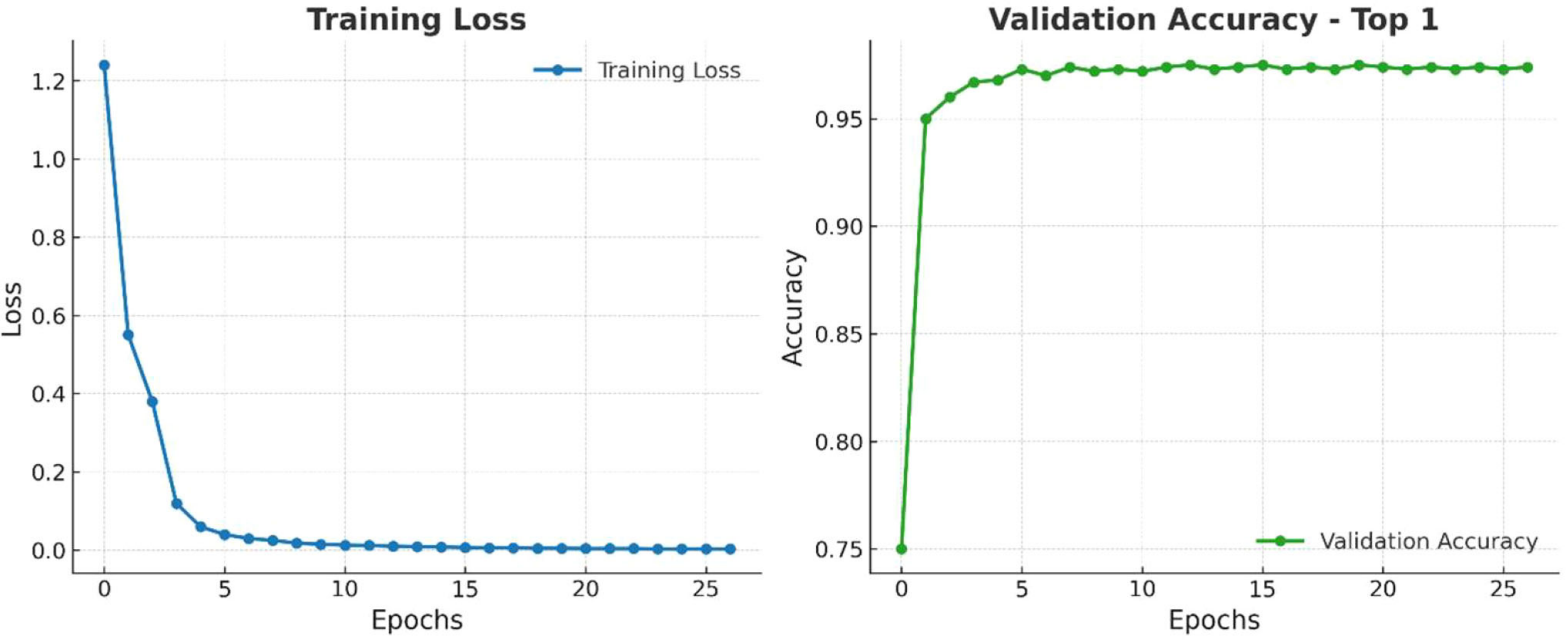
Training loss/validation accuracy of PaLiGemma2 for the detection of date palm disease dataset.

**Figure 20** illustrates three scenarios depicting the prediction ability of PaLI-Gemma2 across three healthy palm leaves. Test Case 1 exhibited 98% confidence of Brown Spots detected by the model; Test Case 2, 95% confidence of a healthy palm leaf; and Test Case 3 exhibited 95% confidence of White Scale detected by the model. These tests yield a robust and reliable model that can generalize across various disease and pest conditions. **Figure 21** provides a view of the model’s learning progress over 30 epochs. Training loss decreased rapidly from 1.2 to near zero in the early epochs, while validation accuracy increased sharply from 75% to above 95%, stabilizing between 96% and 97%, indicating strong generalization, stability, and no overfitting. Overall, PaLI-Gemma2 demonstrated reliability and high accuracy, making it a feasible candidate for use in agricultural diagnostics.

**Figure 22:**
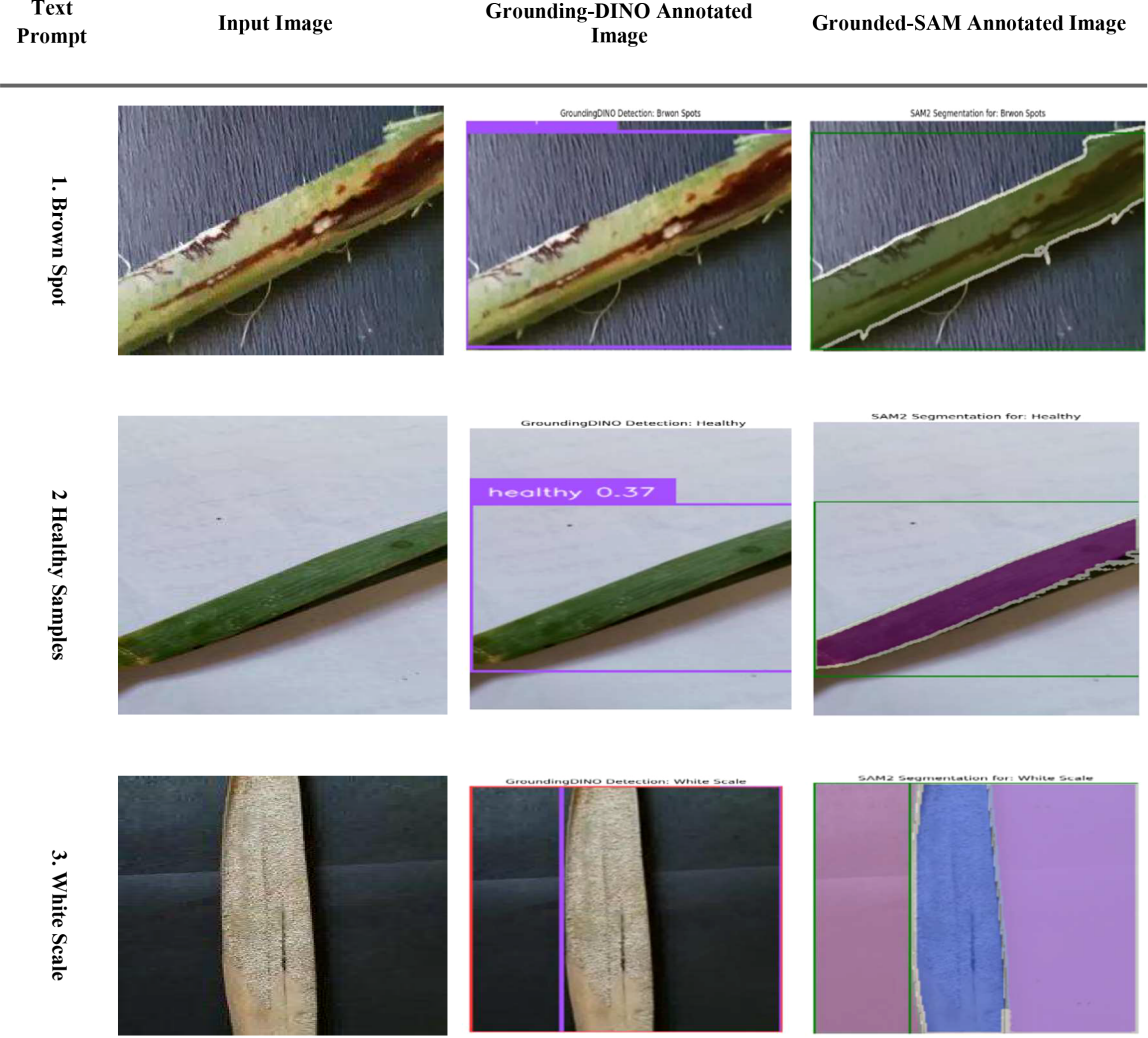
Evaluation of grounding-DINO and grounded SAM for segmentation on date palm disease dataset.

**Figure 22** illustrates the step-by-step process of zero-shot segmentation using Grounding DINO and Grounded-SAM on a date palm disease dataset. Specifically, the process consists of four steps:(1) the text request and input image are loaded sequentially; (2) Grounding DINO identifies all objects relevant to the text request (e.g., “Brown Spot” or “White Scale”) and creates bounding boxes around the objects to be segmented; (3) the bounding boxes generated by Grounding DINO are passed to SAM; and (4) SAM provides high-quality pixel-level segmentation masks, which are displayed on the Grounded-SAM annotated image. This Figure illustrates a straightforward process for transitioning from text-based queries to pixel-level accurate mapping for multiple diseases.

**Figure 23:**
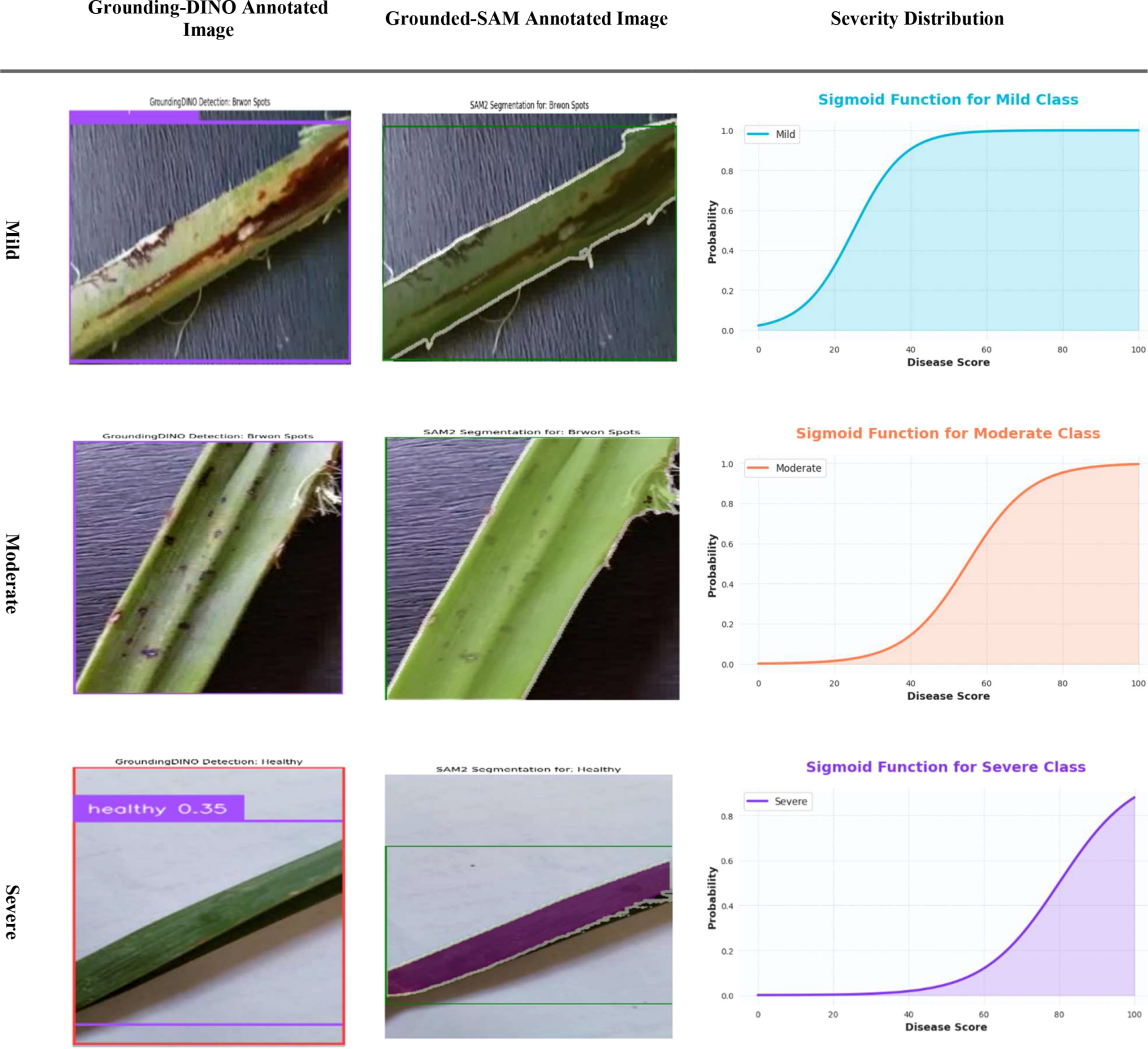
Evaluation of the severity ratio distribution of mild, moderate, and severe on infected date palm leaves dataset.

**Figure 23** provides a thorough and efficient demonstration of the segmentation results by severity. First, the input image is processed by Grounding DINO to obtain a bounding box (“Grounding-DINO Annotated Image”). Next, the bounding box is converted into a simple segmentation mask by SAM (“Grounded-SAM Annotated Image”) to ensure accurate pixel-wise segmentation. Lastly, regarding the sigmoidal functions of Mild, Moderate, and Severe (“Severity Distribution”), these distributions display the probability that a leaf is likely to belong to a certain class of severity based on its “Disease Score.” This evaluation demonstrates a professional and straightforward approach to quantifying and classifying the development of date palm diseases over time.

**Figure 24:**
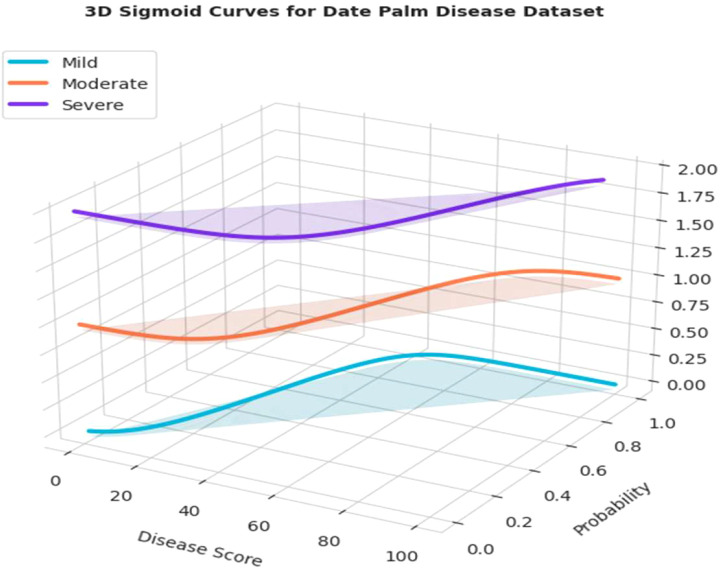
3-D evaluation of severity ratio distribution of mild, moderate and severe date palm disease dataset.

**Figure 24** presents a three-dimensional representation of the severity ratio distribution of the model’s output. The plot does exactly that, it plots “Disease Score” to the probability of a leaf belonging to one of the three severity classes (Mild, Moderate, and Severe). As indicated in the curves, when the disease score increases, the probability of classifying the condition as “Severe” also increases, albeit the chances of being classified as “Mild” and “Moderate” are adjusted accordingly. The general advantage of a 3D representation is that it provides a comprehensive visual representation of the severity distribution, offering a clear, professional interpretation of how the model separates its predictions across different levels of disease progression.

**Table 7** presents a performance comparison of recent deep learning models for date palm disease classification, reporting accuracy, precision, recall, and F1-score. The DenseNet121 + SE Attention model demonstrated the best overall performance, achieving 98.2% accuracy, 95.2% precision, 94.8% recall, and a 97.4% F1-score (Abdallah et al., 2025). This benefit shows the modelling value of incorporating attention mechanisms for more dynamic feature extraction. Using MobileNetV2 (Hassan et al., 2025) for nine-class disease classification yielded an accuracy of 96.9%, with a macro average F1-score of approximately 97%. The high macro average is a demonstration of an overall strong performance; however, no precision and recall metrics were shared. A UAV-based voting ensemble achieved 97.1% accuracy, 94.9% precision, 96.7% recall, and 94.8% F1 score macro averages. This illustrates the benefits of incorporating multiple models to stabilize and balance predictions. Using ResNet & Inception-ResNet transfer learning resulted in 96.9% accuracy, 93.5% precision, and a 94.7% F1-score, although recall was not reported; therefore, it is more challenging to assess recall sensitivity at the class level. In contrast to the baselines, the Overall performance of the Proposed Hybrid Framework across all measurement metrics yields the highest statistics in the case of regress-based appendix classification, with statistics of 98.9% accuracy, 96.5% precision, 96.3% recall, and 95.3% F1-score. Collectively, the improvements can be attributed to the hybrid model consisting of a multifaceted framework with GAN-based data augmentation to improve generalization, CLIP for vision–language alignment, PaLiGemma2 for multimodal reasoning, Grounding DINO for precise object detection, SAM2 for high-quality segmentation, a Vision Transformer (ViT) for global feature representation (i-e sacrosanct), and a regression head for severity classification. The generative and multimodal capabilities of PaLiGemma2 extended the functionality of features in feature space. The detection-segmentation pipeline ensured object localization with spatial precision, and the ViT backbone was able to learn long-range dependencies, making this framework remarkably accurate and robust.

**Table 7:** Assessment of various models’ performance on infected date palm leaves.

Model/technique	Accuracy (%)	Precision (%)	Recall (%)	F1-score (%)
DenseNet121 + SE Attention ^35^	98.2	95.2	94.8	97.4
MobileNetV2 (Disease Classification, 9 Classes) ^36^	96.9	(macro avg)	(macro avg)	~97 (macro avg F1)
Voting Ensemble (UAV-based Multi-class Classification)^35^	97.1	94.9 (macro)	96.7 (macro)	94.8 (macro)
ResNet & Inception-ResNet (Palm Leaf Disease Detection)^1^	96.9	93.5	-	94.7
Proposed Hybrid Framework	**98.9**	**96.5**	**96.3**	**95.3**

The bold values show the proposed framework result to distinguish it from others models results.

**Figure 25:**
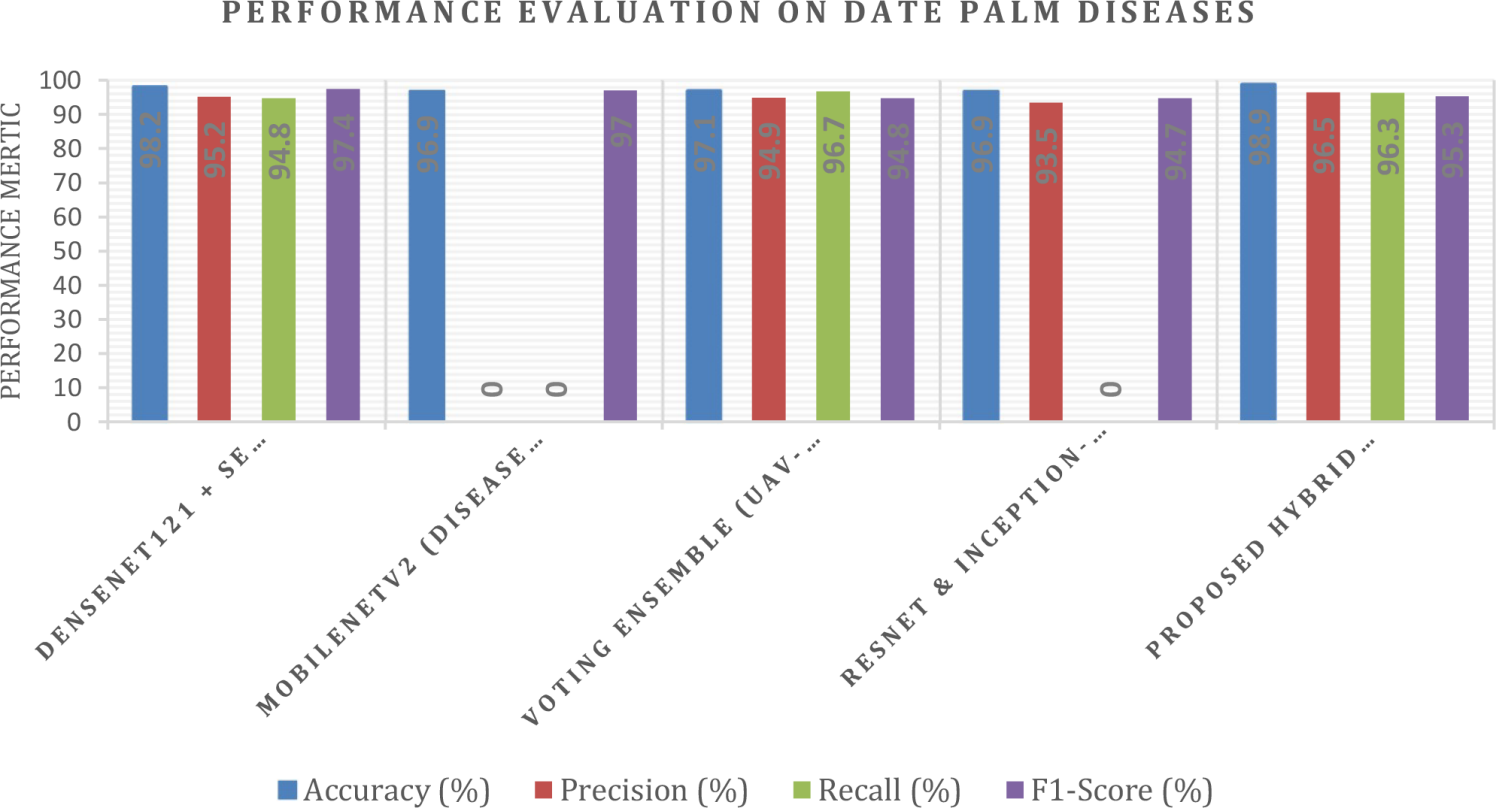
Performance metric of date palm disease dataset.

**Figure 25** contrasts five models for classifying date palm disease in terms of four measures. The proposed hybrid framework achieved the highest outcome, with scores of 98.9% accuracy, 96.5% precision, 96.3% recall, and 95.3% F1-score, marking a performance that surpassed or matched that of all other models. The closest competitor was DenseNet121 + SE Attention, with scores of 98.2% accuracy and 97.4% F1-score.

**Figure 26:**
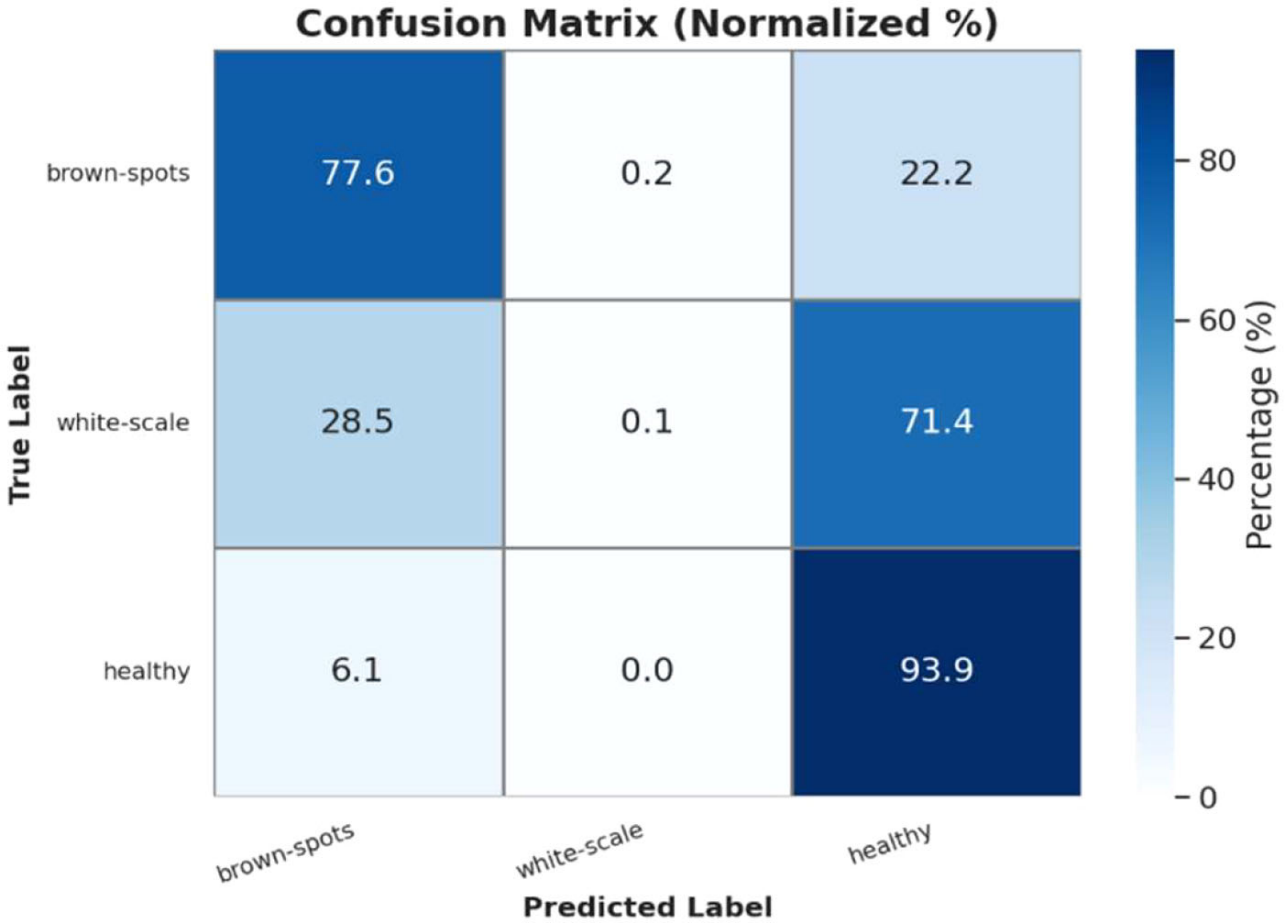
Date palm disease normalized confusion matrix.

Three test cases from the palm leaf dataset are illustrated in **Figure 26** to provide predictive performance of the PaliGemma2 model for three different health conditions. In Test Case-1, the model predicted Brown Spots disease with a 98% confidence, demonstrating that the model can be highly accurate based on consistent visual clues found for each specific disease. In Test Case-2, the model correctly identified a Healthy palm leaf with 95% confidence, demonstrating its ability to differentiate a normal leaf from one infected with disease. The critical aspect here was in Test Case-3, where the model identified a White Scale infestation, and was confident at the 95% interval. The previous two test cases demonstrate that this model can identify the types of diseases and infestations, is robust, accurate, and generalizes well across different health conditions in palm leaves. These examples also provide support for a valid opportunity to deploy a predictive decision model, such as PaliGemma2, as a reliable agricultural diagnostic tool in the field.

## 7 Conclusion and future direction

The presented work proposes a Hybrid Vision-Language and Transformer-based AI framework for comprehensive diagnosis of date palm diseases, comprising GAN-based data augmentation, CLIP-based multimodal classification, PaliGemma2 for text-based detection, Grounding DINO and SAM 2.1 for zero-shot segmentation, and a Vision Transformer regression model to estimate severity based on segmentation validation. The framework demonstrated superior benchmark-based performance on two domain-specific datasets: the nine-class Infected Date Palm Leaves Dataset and the three-class Date Palm Disease Dataset. Notably, the framework achieved an accuracy of 98%, a precision of 95.8%, a recall of 91.3%, and an F1-score of 94.2% and had high accuracy in detection (94-98%) and strong segmentation capability and severity prediction. The systems approach enabled the development of generative AI for dataset balancing, multimodal learning for classification, and prompt-based detection and segmentation, resulting in an explainable end-to-end pipeline that overcomes the limitations of previous unimodal or task-isolated systems. By integrating classification, detection, segmentation, and severity prediction into a single workflow, the framework provides a scalable, accurate, and interpretable approach to agricultural disease management that can be applied to real-world field conditions.

- Looking forward, future work will be focused on several extensions to expand applicability and to enhance robustness:
- Scaling to Multi-Crop Systems: Adapt the framework to other economically important crops and types of diseases to validate its ability to generalize.
- Integration to IoT and UAV Platforms: Move the model to edge devices and drones to implement panoramic, real-time plantation monitoring at scale.
- Measuring Disease Progression over Time: Incorporate time-series data to model the dynamics of disease spread and possible early intervention strategies.
- Active Learning for Annotation Support: Incorporate semi-supervised and active learning approaches to scale the labelled dataset more effectively.
- Improved Explainability: Enhance human capacity to decipher diagnostic reports by utilizing visual-textual reasoning modules designed to create more intuitive, human-interpretable reports for farmers and agronomists.
- Location and Climate-aware Detection: Incorporate environmental and climatic features into the multimodal pipeline to enhance disease detection awareness.

## Data Availability

Publicly available datasets were analyzed in this study. This data can be found here: <b>1)Kaggle Dataset: The date palm leaf disease dataset used for this study is available on Kaggle and can be accessed via the following link: https://www.kaggle.com/datasets/hadjerhamaidi/date-palm-data. This dataset contains images of date palm leaves categorized into three classes: healthy leaves, brown spot disease, and white scale infection. 2)Mendeley Dataset: Additionally, the second dataset, which includes images of date palm leaves with eight types of diseases, can be accessed via Mendeley Data: https://data.mendeley.com/datasets/g684ghfxvg/2. This dataset was collected from 10 date farms in Madinah, Saudi Arabia.
